# Expression profiling of *in vivo* ductal carcinoma in situ progression models identified B cell lymphoma-9 as a molecular driver of breast cancer invasion

**DOI:** 10.1186/s13058-015-0630-z

**Published:** 2015-09-17

**Authors:** Hanan S. Elsarraj, Yan Hong, Kelli E. Valdez, Whitney Michaels, Marcus Hook, William P. Smith, Jeremy Chien, Jason I. Herschkowitz, Melissa A. Troester, Moriah Beck, Marc Inciardi, Jason Gatewood, Lisa May, Therese Cusick, Marilee McGinness, Lawrence Ricci, Fang Fan, Ossama Tawfik, Jeffrey R. Marks, Jennifer R. Knapp, Hung-Wen Yeh, Patricia Thomas, D. R. Carrasco, Timothy A. Fields, Andrew K. Godwin, Fariba Behbod

**Affiliations:** 1 Department of Pathology, University of Kansas Medical Center, 3901 Rainbow Blvd, Mail Stop 3003, Kansas City, KS 66160 USA; 2 School of Medicine, University of Kansas Medical Center, Kansas City, KS 66160 USA; 3 Department of Radiology, University of Kansas Medical Center, Kansas City, KS 66160 USA; 4 Department of Cancer Biology, University of Kansas Medical Center, Kansas City, KS 66160 USA; 5 Department of Biomedical Sciences, University at Albany-SUNY, Rensselaer, NY 12144 USA; 6 School of Public Health, University of North Carolina, Chapel Hill, NC 27599 USA; 7 Department of Chemistry, Wichita State University, Wichita, KS 67260 USA; 8 School of Medicine, University of Kansas, Wichita, KS 67214 USA; 9 Department of Surgery, University of Kansas Medical Center, Kansas City, KS 66160 USA; 10 Department of Radiology, Truman Medical Center, Kansas City, MO 64108 USA; 11 Department of Surgery, Duke University, Durham, NC 27710 USA; 12 Department of Biostatistics, University of Kansas Medical Center, Kansas City, KS 66160 USA; 13 Kansas Intellectual and Developmental Disabilities Research Center, University of Kansas Medical Center, Kansas City, KS 66160 USA; 14 Department of Pathology, Dana-Farber Cancer Institute, Harvard Medical School, Boston, MA 02115-5450 USA

## Abstract

**Introduction:**

There are an estimated 60,000 new cases of ductal carcinoma in situ (DCIS) each year. A lack of understanding in DCIS pathobiology has led to overtreatment of more than half of patients. We profiled the temporal molecular changes during DCIS transition to invasive ductal carcinoma (IDC) using in vivo DCIS progression models. These studies identified B cell lymphoma-9 (BCL9) as a potential molecular driver of early invasion. BCL9 is a newly found co-activator of Wnt-stimulated β-catenin-mediated transcription. BCL9 has been shown to promote progression of multiple myeloma and colon carcinoma. However BCL9 role in breast cancer had not been previously recognized.

**Methods:**

Microarray and RNA sequencing were utilized to characterize the sequential changes in mRNA expression during DCIS invasive transition. BCL9-shRNA knockdown was performed to assess the role of BCL9 in *in vivo* invasion, epithelial-mesenchymal transition (EMT) and canonical Wnt-signaling. Immunofluorescence of 28 patient samples was used to assess a correlation between the expression of BCL9 and biomarkers of high risk DCIS. The cancer genome atlas data were analyzed to assess the status of *BCL9* gene alterations in breast cancers.

**Results:**

Analysis of BCL9, by RNA and protein showed BCL9 up-regulation to be associated with DCIS transition to IDC. Analysis of patient DCIS revealed a significant correlation between high nuclear BCL9 and pathologic characteristics associated with DCIS recurrence: Estrogen receptor (ER) and progesterone receptor (PR) negative, high nuclear grade, and high human epidermal growth factor receptor2 (HER2). *In vivo* silencing of BCL9 resulted in the inhibition of DCIS invasion and reversal of EMT. Analysis of the TCGA data showed *BCL9* to be altered in 26 % of breast cancers. This is a significant alteration when compared to HER2 (*ERBB2*) gene (19 %) and estrogen receptor (*ESR1*) gene (8 %). A significantly higher proportion of basal like invasive breast cancers compared to luminal breast cancers showed BCL9 amplification.

**Conclusion:**

BCL9 is a molecular driver of DCIS invasive progression and may predispose to the development of basal like invasive breast cancers. As such, BCL9 has the potential to serve as a biomarker of high risk DCIS and as a therapeutic target for prevention of IDC.

**Electronic supplementary material:**

The online version of this article (doi:10.1186/s13058-015-0630-z) contains supplementary material, which is available to authorized users.

## Introduction

Ductal carcinoma in situ (DCIS) is a complex pathologic condition in which malignant breast epithelial cells proliferate inside the ducts but do not invade the surrounding stroma. Modern screening technologies have made DCIS a more common diagnosis than in the past. Insufficient understanding of DCIS biology has limited advances in therapy. For example, can a subset of DCIS patients be safely monitored with watchful waiting, as has been adopted for certain prostate cancers in men? As it now stands, a large proportion of patients with DCIS are overtreated, as it is estimated that only 25 − 50 % of cases would progress to invasive cancer over time if left untreated [1–3]. Given the current understanding of DCIS, it remains challenging to reliably stratify DCIS lesions with appropriate sensitivity and specificity to predict progression to invasion [3]. The aim of our study is to identify key molecular mechanisms underlying DCIS progression to invasive ductal carcinoma (IDC) and to assess their potential as future predictive biomarkers of high-risk DCIS. Ultimately, the ability to separate DCIS lesions into high vs low risk will advance the field, our understanding of DCIS and ultimately eliminate overtreatment.

It was generally agreed that the molecular profiles of DCIS and IDC were similar and that the genetic program necessary for invasive progression might already exist in the pre-invasive stages of breast cancer [4, 5]. However, there are other conflicting reports making this area of research worth further exploration. For example, one study suggested that there may be gene dosage changes during a transition from DCIS to IDC [6]. Liao and colleagues found differential genomic copy number aberrations in DCIS with an invasive potential compared to pure DCIS by array comparative genomic hybridization (aCGH) [7]. Another study found amplification of distinct loci restricted to a specific population of cancer cells in 3 out of 13 matched DCIS − IDC pairs [8]. Collectively, these latter studies suggest that unique genomic aberrations in some cancer cells or distinct population of cancer cells may drive DCIS to IDC.

In this study, sequential and temporal changes in gene expression during DCIS invasive progression have been characterized by utilizing two systems: DCIS cell line-derived mouse intraductal (MIND) xenograft models (SUM225 and DCIS.COM) and a tandem DCIS/IDC model that uses samples from patients afflicted with DCIS that are synchronous with IDC within the same breast. Both models involve DCIS non-invasive to invasive transition and provide valuable tools for studying the temporal molecular changes associated with DCIS invasive transition.

The MIND is a novel DCIS in vivo model that has been developed by our group [9]. MIND involves injection of DCIS cell lines or cells derived from primary patient DCIS within the mammary ducts of immunocompromised mice. MIND xenotransplantation is a realistic human DCIS model because it mimics the entire process of DCIS progression, including ductal growth as in situ lesions followed by their invasion as they escape the natural barriers of normal myoepithelial cells and the basement membrane. As previously reported by our group [9], DCIS.COM MIND xenografts generate basal-like lesions and become invasive by 10 weeks post-injection, whereas those generated by the SUM225 cells generate human epidermal growth factor 2 (HER2) over-expressing luminal lesions that invade the myoepithelial layer by 14 weeks. The second model includes tandem DCIS/IDC lesions. The lesions are identified radiologically by an area of clustered microcalcifications adjacent to (contiguous with) an invasive mass and sampled by core biopsy. For these studies, six pairs were collected and analyzed by RNA sequencing for differential gene expression comparing DCIS to the corresponding IDC.

Molecular profiling of both in vivo DCIS progression models revealed a significant increase in BCL9 mRNA and protein expression when comparing non-invasive to invasive lesions in our DCIS cell line MIND xenografts and in five out of six DCIS/IDC tandem lesions. BCL9 is a recently identified Wnt pathway activator, which has been shown to play an important role in transcriptional activity of β-catenin in association with lymphoid enhancer-binding factor 1 (LEF)/T cell specific (TCF) family members [10]. BCL9 has been shown to play a critical role in progression of colorectal cancers and multiple myeloma by activation of Wnt oncogenic signaling [11]. However, the role of BCL9 in mammary gland biology and breast cancer has not been explored previously. In this manuscript, we provide evidence that BCL9 serves as a molecular driver of epithelial mesenchymal transition (EMT) and DCIS invasion by the enhancement of canonical Wnt signaling. Therefore, BCL9 may serve as a potential biomarker of high-risk DCIS, guiding appropriate therapy for these lesions and reducing overall overtreatment of other DCIS lesions that are more indolent. Furthermore, BCL9 promises to serve as a therapeutic target for prevention of IDC.

## Methods

### Animals and animal surgery

Mouse surgery was performed on 8- to 10-week-old virgin female NOD-SCID IL2Rgamma ^null^ (NSG) mice that were either bred or purchased from Jackson Laboratories (Bar Harbor, ME, USA) as previously described [9]. Animal experiments were conducted following protocols approved by the University of Kansas School of Medicine Animal Care.

### Cell culture

DCIS.COM and SUM225 were purchased from Asterand, Inc. (Detroit, MI, USA) in 2007 and were maintained according to the supplier’s guidelines. Both cell lines have been authenticated by genomic profiling validating the estrogen receptor-negative, progesterone receptor-negative, HER2-positive (ER^-^ PR^-^ HER2^+^) status of the SUM225 cells and the ER^-^ PR^-^ HER2^-^ expression pattern in the DCIS.COM [12].

### Tandem lesion biopsies

All human experiments were approved by the University of Kansas Medical Center Institutional Review Board (IRB). All patients gave written informed consent for participation in this research. Subject recruits included patients undergoing image-guided core needle biopsy due to suspicion of DCIS or IDC. In all cases, research specimens were obtained only after acquisition of diagnostic specimens. Our radiologists apply minimally invasive ultrasound-guided selective tissue harvest of contiguous lesions with a tandem appearance and provide us with biopsy cores from each region. Biopsy tissue was placed in preservation media (LiforCell, *Life*blood Medical, Inc., Freehold, NJ, USA) and stored at 4 °C on ice until RNA isolation.

### Patient samples for analysis of BCL9 as a potential biomarker of high risk

Tissue sections for BCL9 analysis were provided by Dr. Jeffrey Marks (Duke University) as a part of the NIH Early Detection Research Network (EDRN) GYN/Breast Working Group Initiative to validate biomarkers that may predict a greater risk of invasive breast cancer or worst-prognosis disease. These samples were identified, procured, and sectioned, stored and maintained under a Duke-approved protocol (eIRB Pro00027811, J Marks, PI). Two categories were defined: cases of DCIS that progressed to invasive cancer in the same breast between 1.8 and 17.6 years after initial diagnosis and controls with DCIS that did not progress (either recurrent DCIS or invasive cancer) over a minimum of 10 years follow up. Controls were further selected based on size and nuclear grade to match or exceed the sizes in the cases. The Van Nuys index [13] for controls had a higher median value than the cases (*p* = 0.04).

### RNA isolation, quantitative PCR (qPCR) and wnt target qPCR arrays

Total RNA was isolated with miRNeasy Mini Kit (Qiagen #217004, QIAGEN Inc., Valencia, CA, USA) using the manufacturer’s protocol, and cDNA was synthesized from 250 ng of total RNA with miScript Reverse Transcription Kit (Qiagen #218061). TaqMan® Gene Expression Master Mix (Applied Biosystems #4369016) and TaqMan® Gene Expression assays were used. Primers specific for human BCL9 (Applied Biosystems #Hs00979216_m1) were utilized and target gene expression was normalized to human β-actin (Applied Biosystems #Hs99999903_m1). The standard curve method was used for quantification. Reactions were performed in the StepOnePlus™ Real-Time PCR system and software (Applied Biosystems, part of Thermo Fisher Scientific, Waltham, MA, USA) in 96-well plates. The data were analyzed using the ΔΔ cycle threshold (CT) method [14].

### Statistical analysis

Data are presented as mean normalized expression ± standard error of the mean (SEM). Unless otherwise noted, one-way analysis of variance (ANOVA) was used for statistical comparisons. A value of *p* ≤0.05 was considered significant.

### Microarray gene expression profiling and analysis

We utilized DCIS MIND models, a novel model developed in our laboratory, which most closely mimics the human DCIS environment, with both SUM225 and DCIS.COM cell lines to characterize the sequential and temporal changes in mRNA expression over a time course of 2, 6, and 10 weeks during in vivo progression in the epithelial cells. Microarray technology was utilized to analyze gene expression profiles from RNA isolated from magnetically sorted epithelial cells from MIND xenografts at 2, 6 and 10 weeks post-injection. For these studies, five mice per replicate (three replicates) per time point (three time points; 2, 6, and 10 weeks) for each cell line (two cell lines; DCIS.COM and SUM225) were used. The mammary epithelial cells were magnetically sorted from five mice at each time point per replicate. After sorting, Qiazol extraction of total RNA was performed according to the manufacturer’s instructions. Labeling was performed using the GeneChip 3' IVT Express Kit (Affymetrix, Santa Clara, CA, USA), which utilizes an oligo dT-based reverse transcription reaction followed by a T7 promoted in-vitro transcription biotin labeling reaction. Hybridization was performed using the GeneChip Hybridization, Wash and Stain Kit (900720). The platform used is HG-U133_Plus_2 Affymetrix Human Genome U133 Plus 2.0 Array. GeneChips were scanned using the Affymetrix GeneChip Scanner 3000 7G. Raw mRNA expression values from the 2-week, 6-week and 10-week samples were normalized and converted to the log2 scale. Data were median-centered and analyzed by unsupervised average-linkage hierarchical clustering using Cluster 3.0 software [15]. The computed data matrix was then uploaded into Java TreeView software and visualized as a heat map [16]. Clustering of expression data from DCIS.COM and SUM225 cell lines revealed that the majority of expression changes had already occurred at the 6-week time point with little change occurring between 6 and 10 weeks. This suggests that mechanisms of invasion are already in place by week 6. Further analysis was focused on the 2-week to 6-week time point.

Significance analysis for microarrays (SAM) software was utilized to determine differentially expressed genes between the 2-week and 6-week time point in both DCIS.COM and SUM225 cell lines [17]. The cutoff for significance was determined by <5 % false discovery rate (FDR). Two-class unpaired SAM analysis generated a list of significant genes and fold-change values between 2 and 6 weeks in DCIS.COM (18,590 downregulated; 10,227 upregulated) and SUM225 (19,953 downregulated and 14,691 upregulated). These genes were further analyzed using QIAGEN Ingenuity® Pathway Analysis (IPA®, QIAGEN Redwood City, [18]). IPA software integrates expression changes with known molecular interactions and disease processes [19]. The Wnt/β-catenin canonical pathway was identified as a significantly upregulated pathway in both cell line MIND xenografts during transition from 2 to 6 weeks. The raw and analyzed microarray data have been deposited in the NCBI Gene Expression Omnibus and are accessible through GEO [GEO:GSE65890] [20].

### RNA sequencing and analysis

Total RNA was prepared using the All prep Qiagen Kit, according to the manufacturer’s protocol. Libraries were prepared by illumina TruSeq RNA Sample Preparation Kit (A cat#FC-122-1001, B cat#FC122-1002) according to the manufacturer’s protocol. Libraries were prepared using illumina TruSeq RNA Sample Preparation Kit (A cat#FC-122-1001, B cat#FC122-1002) according to the manufacturer’s protocol. The DCIS and IDC samples were sequenced using a HiSeq2500 2 × 100 bp version 3 sequencing run. Eight sample libraries were multiplexed, four per lane resulting in 46.9X – 58.2X coverage per sample. Four sample libraries were multiplexed, two per lane resulting in 107.4X – 124X coverage per sample. Paired Fastq sequence files were imported to CLC Genomics Workbench (version 7.5) and mapped to the human reference genome (hg19) using the approach previously described [21]. The Ensembl database (GRCh37.74.gtf) was used for gene annotation. Total number of reads mapped to the gene was used as the total counts for the gene, and the values were transformed by adding 1 followed by log2 transformation. Transformed data were quantile-normalized before the analysis of differential gene expression between two groups (DCIS vs IDC). Empirical analysis of differential gene expression was performed between two groups (DCIS vs IDC) using the exact test as previously described [22].

The raw and analyzed RNA sequencing data have been deposited in the NCBI Gene Expression Omnibus and are accessible through GEO [GEO:GSE66301] [23].

### The Cancer Genome Atlas (TCGA) data analysis

All TCGA RNASeqV2 breast cancer data (*rsem.genes.results) were downloaded from TCGA data portal [24]. In R [22, 25–26] the raw counts were normalized for at least 5 counts in at least 113 (number of normal samples). The normalized counts for the gene BCL9 were obtained and log transformed. All cancer samples with BCL9 levels above the range defined by normal samples were labeled UP regulated in cancer (414). Differential gene expression was performed between cancer samples with a normal range of BCL9 and samples with upregulated BCL9. A list of significant genes (1,756 downregulated, 980 upregulated) was obtained with a threshold of FDR ≤0.05 and log fold change 0.26. The significant genes were analyzed in IPA and the Canonical Wnt with a *p* value ≤0.05 was shown to be among the significantly altered pathways.

### Immunofluorescence staining (IF)

IF was performed as previously described [27]. Antibodies are listed in Additional file 1: Table S1. Nuclei were counterstained with 4′,6-diamidino-2-phenylindole (DAPI; Vector Laboratories, # H-1200, Vector Laboratories, Inc, Burlingame, CA, USA). Negative controls were carried out using secondary antibodies without the primary antibodies. Imaging was performed on a laser-scanning confocal microscope (Model 510; Carl Zeiss MicroImaging, Inc, Thornwood, NY, USA). The acquisition software used was Pascal (Carl Zeiss MicroImaging, Inc). Fluorescence quantitation and analysis was done using ImageJ [28]. Images were analyzed for area of selection, mean gray value, and integrated density. Both the areas of interest and their background were measured, then the corrected total cell fluorescence (CTCF) was calculated by the following formula:

$$ \mathrm{CTCF} = \mathrm{Integrated}\ \mathrm{density}\hbox{--} \mathrm{Area}\ \mathrm{of}\ \mathrm{selected}\ \mathrm{cells} \times \mathrm{Mean}\ \mathrm{fluorescence}\ \mathrm{of}\ \mathrm{background}\ \mathrm{readings}. $$

### Plasmids, transfection, and luciferase reporter assay

Plasmid constructs: pSuper8X–TOPFlash reporter (Addgene plasmid 12456, Addgene, Cambridge, MA, USA) and Super 8x FOPFlash (TOPFlash mutant) (Addgene plasmid 12457) were provided by Randall Moon via Addgene [29, 30]. Renilla luciferase plasmid phRG was from Promega. The β-catenin△N (Addgene plasmid 19288) construct was acquired from Eric Fearon via Addgene [30]. PCDH-BCL9 (BCL9-OE), PLKO.1-BCL9-shRNA (BCL9KD) (CCTCTGTTGAATATCCCTGGAA) and PLKO.1-non-silencing control (Control) were acquired from Dr. Carrasco [11]. pGIPZ Human BCL9 shRNA (BCL9 KD 2) (TGCAAACTTGGACATTCGA), and pGIPZ-non-silencing control (Control 2) were obtained from Dharmacon (#RHS4430-200265260). Transfection: DCIS.COM and SUM225 were transfected with electroporation using Amaxa ™ Cell line Nucleofector kit V (Lonza #VCA-1003, Lonza Group Ltd, Basel, Switzerland), while HEK293T cells were transfected using Lipofectamine 2000 reagent (Invitrogen #11668-027, Invitrogen, part of Thermo Fisher Scientific, Waltham, MA, USA) according to manufacturer’s protocols. Luciferase assay was performed using Dual-Luciferase® Reporter Assay System (Promega #E1910, Promega Corporation, Madison, WI ,USA).

### Lentivirus production

Glycerol stocks of pLKO.1 shRNA-based BCL9 and PLKO.1 non-silencing control were cultured in the presence of 100 μg/ml of ampicillin (Amresco # 0339, AMRESCO LLC, Solon, OH, USA) and plasmids were purified using HiSpeed Plasmid midi kit (Qiagen #12643). Preparation of viral particles were performed by co-transfecting individual pLKO.1 vectors (10 μg), packaging plasmid pCMV-dR8.2 (contains Gag, Pol, Rev, and Tat) (Addgene plasmid 8455; 5 μg), and the envelope plasmid pCMV-VSVG (Addgene plasmid 8454; 5 μg) in HEK293T cells. Both plasmids were acquired from Robert Weinberg via Addgene [31]. Transfection was performed in a 10-cm plate, nearly 75 % confluent using Lipofectamine 2000 transfection reagent (Invitrogen #11668-027) in antibiotic free Opti-MEM media (Invitrogen #51985-034) following the manufacturer’s protocol. Media was collected after 48, 72, and 96 h of transduction, pooled and subjected to ultracentrifugation at 80,000 × g for 2 h (Beckman Coulter, Optima L-100 XP, 70Ti rotor). The pellets of the concentrated viral particles were re-suspended in 250 μl of DMEM (GIBCO #21063029, GIBCO, part of Thermo Fisher Scientific, Waltham, MA, USA), and stored in aliquots at −80 °C until further use. Lentiviral titers were measured using Lenti-X™ p24 Rapid Titer kit (Clontech #632200, Clontech, Mountain View, CA, USA). Transduction was performed at an Multiplicity of infection (MOI) of 3 and 20 for DCIS.COM and SUM225, respectively, and cells continued to grow in the presence of puromycin (Thermo Scientific #100552, Thermo Scientific, part of Thermo Fisher Scientific, Waltham, MA, USA). For various experiments, transduced cells from up to five passages were used.

### MTS, invasion and migration assays

For MTS assays, Cell Titer 96® Aqueous Non-Radioactive Cell Proliferation Assay (Promega #G5421) was used according to the manufacturer’s protocol. Transwell assays were used to measure invasion and migration. The upper side and underside of the transwells (Corning #3422, Corning, Inc, Corning, NY, USA) were coated with Corning™ Matrigel™ Membrane Matrix (Fisher Scientific #CB-40230) (1 mg/ml in serum-free media) for the invasion assay, and with Corning™ Human Fibronectin (Fisher scientific #CB-40008, Fisher Scientific, part of Thermo Fisher Scientific, Waltham, MA, USA) (50 μg/ml PBS/0.1 % gelatin) for the migration assay. DCIS.COM and SUM225 cells were starved of serum for 24 h prior to use. Cells were irradiated at 10 gray and 2.5 × 10^4^ cells were plated in serum-free media in the upper well. The percent area of migration and invasion were analyzed in DCIS.COM and SUM225 cells after 24 and 96 h, respectively. Invasion and migration were determined by ImageJ [28] analysis of microscopic images.

For in vivo invasion studies, BCL9-KD, non-transduced (NT), and scrambled shRNA control (control) SUM225 and DCIS.COM cells were injected at 10,000 cells per gland. A total of three glands and three animals in control groups, five glands and four animals in KD groups and four glands in four animals in NT groups were examined. The glands were collected at 10 and 14 weeks post-intraductal injection in DCIS.COM and SUM225 xenografts, respectively. The mammary glands containing DCIS-like lesions were then fixed, embedded and sectioned at 5 μm. Every tenth section was stained with H&E to identify the sections with the greatest xenograft growth for each gland. Then four sections adjacent (two sections on each side) to the one with the greatest growth were prepared for IF as described above, stained for human-specific K5/K19, SMA and counterstained with DAPI. Imaging was performed as described above. Invasive lesions were identified by the lack of a smooth muscle actin (SMA)-expressing myoepithelial layer. The LSM image browser was used to measure the maximum distance of an invasive lesion to the closest DCIS lesion in each section. To determine the number of invasive lesions per section, confocal images (×20 magnification) were taken of all invasive lesions and counted. Measurements (i.e., distance of invasion and number of invasive lesions) for the four sections were averaged to represent each gland. Data are presented as the maximum distance of invasion (μm) and number of invasive areas in each section.

### Western blot analysis and co-immunoprecipitation

For co-immunoprecipitation, 1,000 μg of protein was incubated with antibodies at 4 ^o^C overnight followed by centrifugation at 3,000 rpm for 1 minute in 4 ^o^C. The supernatants were incubated with Protein A/G PLUS-Agrose beads (Santa Cruz #sc-2003, Santa Cruz Biotechnology, Inc., Dallas, Texas, USA) at 4 °C for 1 h, followed by a wash in PBS. Proteins bound to the beads were eluted with SDS-loading buffer at 99 °C for 5 minutes and then loaded for western blot, and 2 μg of whole cell lysates were loaded as input. Western blots analysis was carried out as previously described [32]. For western blots, 25 μg of DCIS.COM and 50 μg of SUM225 cell lysates were loaded into each lane. The antibodies used are listed in Additional file 1: Table S1.

### FACS analysis and magnetic sorting

The cells were stained at a final concentration of 1∶20 for 30 minutes on ice followed by washes in Hanks’ Balanced Salt Solution (Invitrogen #24020-117) containing 2 % fetal bovine serum. The antibodies used are listed in Additional file 1: Table S1. FACS and data analysis were performed using the BD LSR II flow cytometer and FlowJo software (Tree Star). Magnetic sorting was performed using Easy Sep® human Epcam positive selection kit (Stem Cell Technology #18356, STEMCELL Technologies Inc., Vancouver, BC, Canada) according to the manufacturer’s protocol.

## Results

### BCL9 upregulation is associated with DCIS epithelia that progress to invasion

**Fig. 1:**
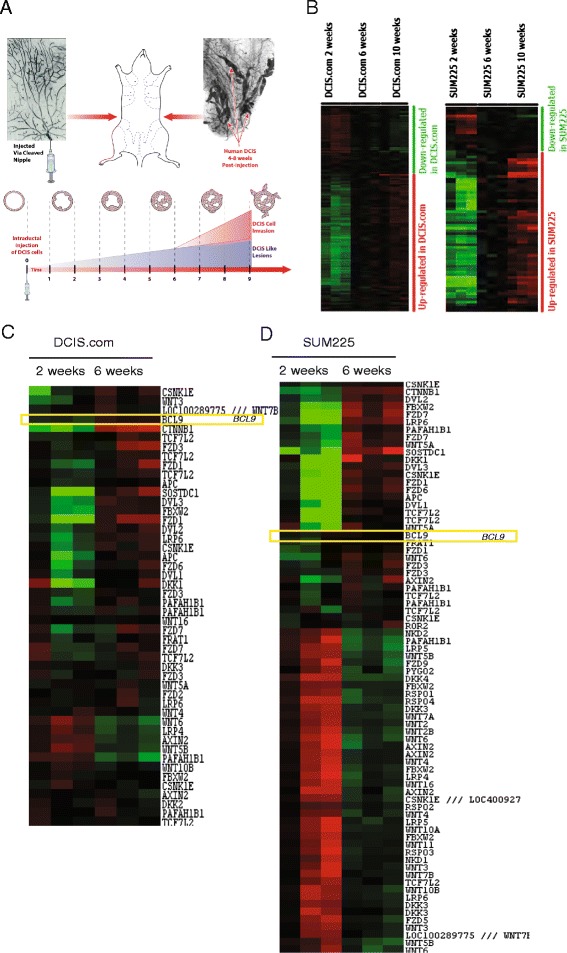
Differentially expressed genes in DCIS.COM and SUM225 mouse intraductal xenograft models (MIND). **a** The MIND model. MIND xenografts were generated by intraductal injection of DCIS.COM and SUM225 cells into the mammary ducts of immunocompromised mice. Mammary glands containing DCIS-like lesions were collected at the distinct stages of in situ to invasive lesions (2, 6 and 10 weeks) followed by digestion, magnetic sorting of epithelial cells and RNA isolation. The RNA was subjected to microarray analysis. **b** Heatmap of differentially expressed genes in DCIS.COM and SUM225 MIND xenografts at 2, 6 and 10 weeks. Unsupervised average-linkage hierarchical clustering of expression data from DCIS.COM and SUM225 MIND xenografts revealed that the majority of expression changes occurred at the 2-week to 6-week time point with little change occurring from 6 to 10 weeks. Further analysis was focused on the 2-week to 6-week time points. **c**, **d** Heatmap of differentially expressed genes in the canonical Wnt pathway from 2 to 6 weeks in DCIS.COM (**c**) and SUM225 (**d**) MIND xenografts. Unsupervised average-linkage hierarchical clustering was used to visualize significantly upregulated or downregulated genes in the WNT pathway, using a cutoff false discovery rate of <5 %

**Fig. 2:**
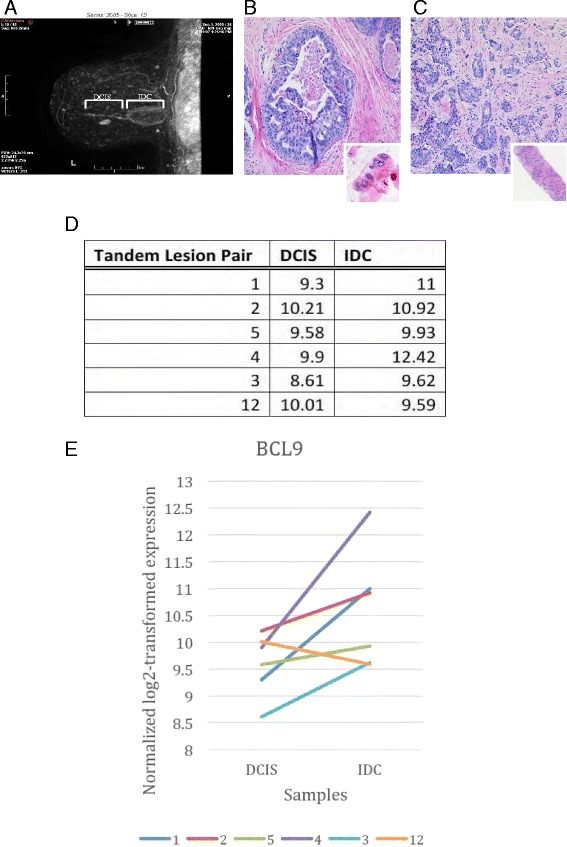
Analysis of RNA sequencing data of tandem lesions for BCL9 expression comparing DCIS to invasive ductal carcinoma. Radiographic (**a**) and H&E stain (**b**, **c**) images of a patient’s DCIS/IDC tandem lesion. **a** Three-dimensional ultrasound image of a tandem lesion. **b** H&E staining of a biopsy taken from the DCIS and (**c**) the IDC regions. Insets: lower magnification in **b**, **c**, respectively, demonstrating that the biopsies were highly pure and composed of either DCIS or IDC. **d**, **e** Normalized log2 transformed expression of BCL9 was plotted and tandem lesions between paired DCIS and IDC are connected by a line to indicate their relationship. The results indicate a significant increase in BCL9 expression in IDC samples compared to the DCIS lesions (*p* = 0.01)

In order to explore the temporal molecular changes associated with DCIS non-invasive to invasive transition, we utilized our DCIS cell line MIND models and DCIS/IDC tandem lesions. The mammary glands containing DCIS-like lesions were excised followed by digestion to isolate the epithelial cell components at three time points: 2, 6 and 10 weeks post-injection. These time points were selected in order to accurately reflect the molecular changes, as the DCIS lesions are formed between 2 and 6 weeks and progressed past the myoepithelial layer and the basement membrane by 10 weeks (Fig. 1a). In order to separate human DCIS epithelial cells from mouse mammary cells, EpCAM-positive cells were magnetically sorted followed by RNA isolation and microarray analysis. The majority (>90 %) of DCIS.COM and SUM225 cells express EpCAM, Additional file 2: Figure S1. Additionally, we performed RNA sequencing of patient DCIS/IDC tandem lesion pairs. A heatmap of analyzed microarray data is shown in Fig. 1b-d, and the differentially expressed genes are shown in Additional file 3. We focused our analysis on the canonical Wnt signaling, because a recent report by Scheel and colleagues demonstrated that the collaboration of three signaling pathways, transforming growth factor beta (TGF-β), canonical and non-canonical Wnt signaling induced and maintained an EMT state in mammary epithelial cells [33]. Acquisition of an EMT-like phenotype is believed to be the initiating event prior to cell invasion. An EMT-like phenotype can result from an aberrant basal differentiation program in differentiated luminal/epithelial cells or in stem/progenitor cells [34]. Furthermore, this study showed that pre-treatment of epithelial cells with the Wnt activators followed by TGF-β and downregulation of E-cadherin resulted in a synergistic enhancement in EMT and cellular migration. These data suggest that Wnt signaling is the earliest event in the process of EMT and cellular invasiveness. We decided to concentrate on one of the genes in the canonical Wnt signaling pathway, B cell lymphoma-9 (*BCL9*), because it was found to serve as a co-factor of β-catenin in early 2000 [35], however, there were no previous studies on the role of BCL9 in breast cancer. BCL9 is located on chromosome 1q21, a common amplified region in breast cancer. Analysis of the microarray data of the MIND samples showed canonical Wnt signaling to be among the significantly upregulated pathways in our dataset (Fig 1c-d, Additional file 4) and BCL9 was significantly upregulated during the transition from DCIS to IDC (*q* value <5 %) (Fig. 1c-d and Additional file 3). We also assessed BCL9 expression in six pairs of DCIS/IDC tandem lesions by RNA sequencing. Tandem DCIS/IDC are defined as DCIS lesions that have concurrent IDC within the same breast (Fig. 2a-c). The analysis of tandem lesion RNA sequencing data for BCL9 expression comparing DCIS to IDC is shown in (Fig. 2d-e). This analysis showed a significant upregulation in BCL9 expression in the IDC component compared to DCIS (Fig. 2a-e).

**Fig. 3:**
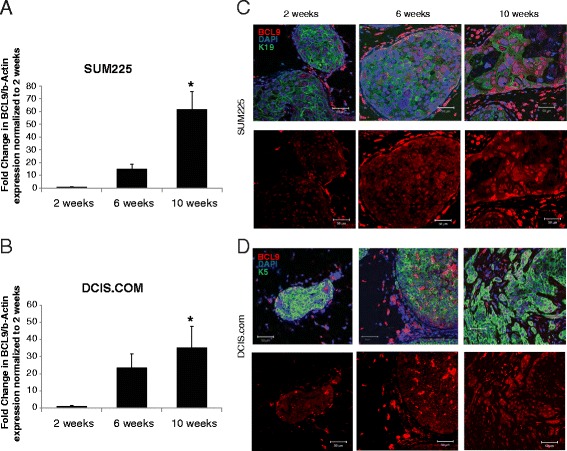
Enhanced BCL9 nuclear expression in DCIS cell line MIND xenografts that progressed to invasive lesions. **a**, **b** RT-qPCR of BCL9 in epithelial cell adhesion molecule (EpCAM)-positive epithelial cells sorted from SUM225 (**a**) and DCIS.COM (**b**) MIND xenografts at 2, 6, and 10 weeks post-intraductal injection. Bar graphs represent fold change normalized to 2 weeks. Data are mean values ± standard error of the mean (n = 3, *p <0.05). **c**, **d** Immunofluorescent (IF) staining of BCL9, keratin (K)5/K19, and 4′,6-diamidino-2-phenylindole (DAPI) in SUM225 (**c**) and DCIS.COM (**d**) MIND xenografts at 2, 6, and 10 weeks post-intraductal injection; merged channels are shown in the *upper panels* and BCL9-only channels are shown in the *lower panel*. BCL9 was conjugated to Alexa-Fluor 594 (*red*) and keratin 5/keratin 19 were conjugated to Alexa-Fluor 488 (*green*). Nuclei were counterstained with DAPI. Scale bars are 50 μm; × 40 objective was used

To confirm our microarray analysis, RT-qPCR was performed on EpCAM-positive cells sorted from an independent set of DCIS cell line MIND xenografts as they progressed from 2 to 10 weeks. BCL9 gene expression was significantly increased at 10 compared to 2 weeks in both SUM225 and DCIS.COM MIND xenografts (62 ± 14 and 35 ± 12 fold increase, respectively; mean ± SEM *p* <0.05) (Fig. 3a, b). Furthermore, IF staining of the MIND xenografts demonstrated increased nuclear BCL9 expression as DCIS lesions progressed to invasion (Fig. 3c, d). There have been a few reports on the role of BCL9L (BCL9-2 or B9L), BCL9 homolog, in breast cancer. One study showed nuclear BCL9L expression to be significantly associated with high nuclear grade and the expression of HER2 in breast cancers [36]. Another study reported that BCL9L induced ER positive breast cancers in vivo by regulating the expression of ER through a β-catenin independent mechanism and predicted therapeutic response to tamoxifen [37]. The human *BCL9* and its homolog *BCL9L* reside on chromosome 1q21 and 11q23.3 respectively. Both BCL9 and BCL9L have been shown to function as co-activators of β-catenin-LEF/TCF mediated transcription [38, 39]. We compared the expression patterns of BCL9 and BCL9L in DCIS cell line MIND xenografts and on tissue sections obtained from 23 patients with DCIS and associated IDC and 14 patients with pure DCIS. Additional file 5: Figure S2A shows that BCL9L expression was mainly cytoplasmic, while BCL9 expression was primarily nuclear. Furthermore, RT-qPCR showed no significant increase in BCL9L expression in DCIS MIND xenografts during invasive transition from 2 to 10 weeks (Additional file 5: Figure S2B-C). Western blot on cell lysates obtained from DCIS cell lines also showed no change in BCL9L expression with BCL9 KD. Therefore, the results in both the MIND and tandem lesions support the hypothesis that increased BCL9 expression is associated with DCIS transition to invasion, while our data do not show a change in BCL9L expression associated with DCIS progression.

### BCL9 knockdown (KD) inhibits the proliferative, migratory, and invasive activity of DCIS cells in vitro and in vivo

The canonical Wnt pathway is required for normal development and tissue homeostasis [40]. However, aberrant activation of canonical Wnt signaling has been implicated in the development and progression of many cancers including breast cancer [41, 42]. BCL9 overexpression has been proposed as one mechanism that may contribute to the aberrant Wnt activation [11]. BCL9 possesses a potent transcription activation domain and might function as an oncogene by providing an alternative pathway for β-catenin activation and subsequent tumor progression [35].

**Fig. 4:**
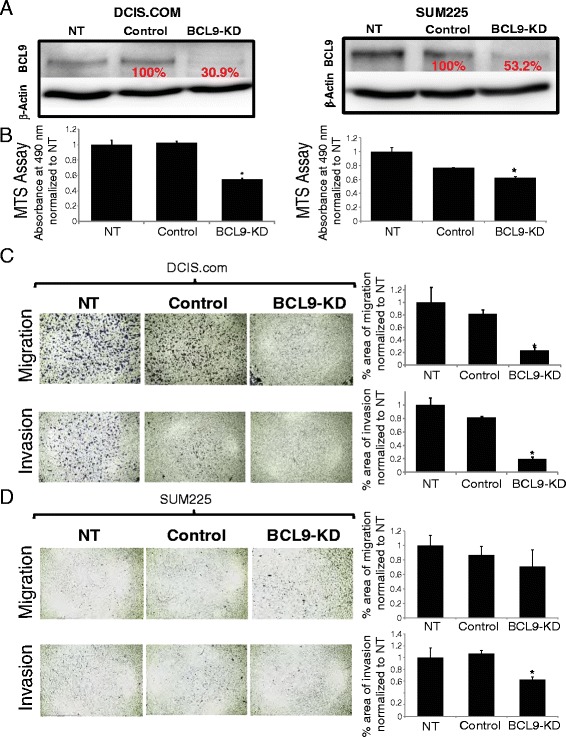
BCL9-KD decreased proliferative, migratory and invasive activity of DCIS cell lines in vitro. **a** Western blot analysis using anti-BCL9 antibody and anti-β-actin as a loading control (*top*), and (**b**) MTS assays of non-transduced (NT), scrambled control (*control*) and BCL9-KD in DCIS.COM (*left panels*) and SUM225 (*right panels*). Bar graphs represent mean absorbance at 490 nm normalized to NT ± standard error of the mean (SEM) (n = 3, * *p* <0.05). **c**, **d** Representative images of the migration and invasion assays. Bar graphs represent percent area of cells migrated (*top*) and invaded (*bottom*) under the membrane after 24 h for DCIS.COM and 96 h for SUM225. Invasion and migration were determined by ImageJ analysis of microscopic images per sample, the data are mean values normalized to NT ± SEM (n = 3, * *p* <0.05)

To assess the role of BCL9 in promotion of DCIS invasive progression, two shRNA-based BCL9 constructs have been utilized: shRNA1 [11] and shRNA2, as well as their corresponding scrambled controls (Control 1 and Control 2). As the values for NT and scrambled shRNA (control) were similar in all of the experiments, only the values for the shRNA control groups are listed in the results section. Western blot confirmed that shRNA1 efficiently knocked down BCL9 in both DCIS.COM (Fig. 4a; left panel) and SUM225 (Fig. 4a; right panel). We have also demonstrated efficient BCL9 KD using shRNA2 (Additional file 6: Figure S3A). MTS assay was performed to assess the role of BCL9 on cell growth in vitro (Fig.4b and Additional file 6: Figure S3B). As shown in Fig. 4b, BCL9 KD significantly suppressed growth by 0.55 ± 0.01 fold (*p* <0.05; compared to 1.02 ± 0.02 in control) in DCIS.COM and by 0.62 ± 0.01 fold in SUM225 (*p* <0.05; compared to 0.76 ± 0.004 in control). To assess the role of BCL9 on cell migration and invasion, fibronectin and reconstituted basement membrane (Matrigel) assays were performed, respectively (Fig. 4c, d). BCL9 KD reduced invasion of DCIS.COM cells (0.23 ± 0.03 fold, *p* <0.05) compared to control (0.81 ± 0.07), and in SUM225 cells (0.62 ± 0.04 fold, *p* <0.05) compared to control (1.06 ± 0.05). In addition, cell migration in DCIS.COM BCL9 KD was significantly lower (0.2 ± 0.03 fold, *p* <0.05) compared to control cells (1.00 ± 0.11, *p* <0.05). However, there was no significant reduction in SUM225 BCL9 KD (0.70 ± 0.23 fold, *p* <0.05) migration compared to the control (1.00 ± 0.14 fold). The results for MTS assay have been confirmed using shRNA2 (Additional file 6: Figure S3B) and for invasion and migration (Additional file 6: Figure S3C-D). While there was a trend towards a reduction in migration and invasion for SUM225 using shRNA2, these results did not reach statistical significance (Additional file 6: Figure S3D). Furthermore, re-expression of BCL9 in BCL9 KD DCIS.COM cells using a BCL9-overexpression lentiviral vector, resulted in a significant increase in proliferation, migration and invasion in vitro (Additional file 7: Figure S4A-D) (*p* <0.05).

**Fig. 5:**
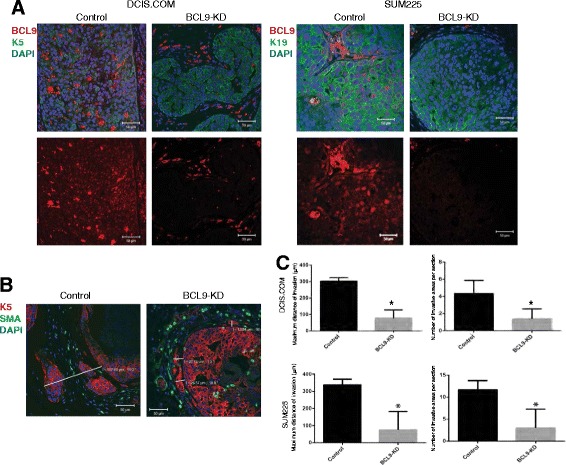
BCL9-KD inhibits invasion in DCIS cell line mouse intraductal xenograft model (MIND) xenografts. **a** Immunofluorescent (IF) staining of BCL9 (*red*), K5/K19 (*green*), and 4′,6-diamidino-2-phenylindole (DAPI) (*blue*) in DCIS.COM (*left*) and SUM225 (*right*), control and BCL9-KD MIND xenografts at 10 and 14 weeks post-intraductal injection, respectively. **b** IF staining of K5 (*red*), smooth muscle actin (SMA) (*green*), and DAPI in DCIS.COM cells demonstrating how distance of invasion was measured. Scale bars = 50 μm, × 40 magnification. **c** Bar graphs represent the maximum distance of invasion and number of invasive lesions in control and BCL9-KD for DCIS.COM and SUM225 MIND xenografts. Data represent the mean ± standard error of the mean (n = 4, *p <0.05)

**Fig. 6:**
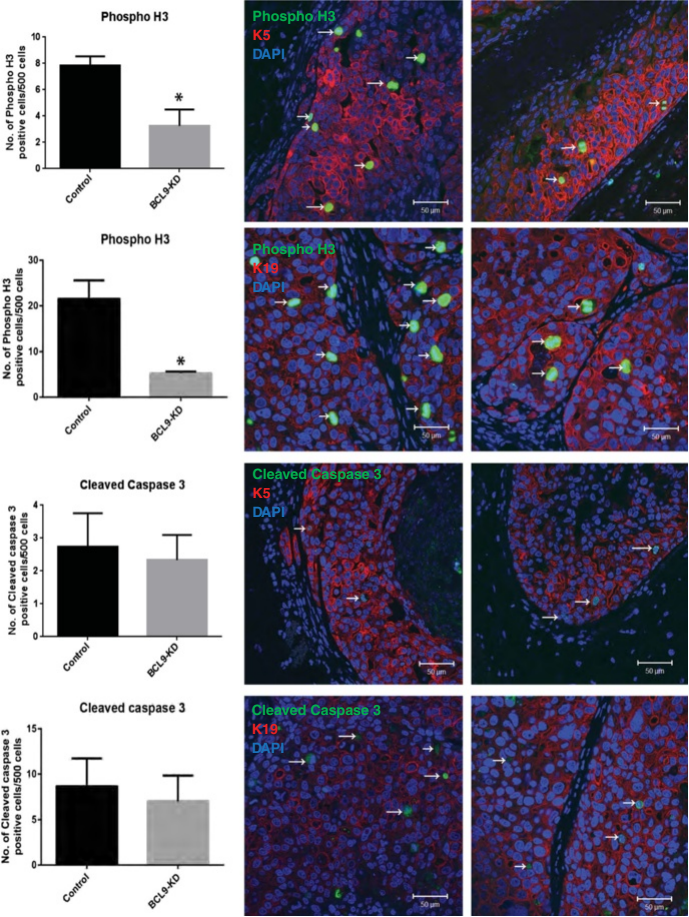
BCL9-KD decreased epithelial cell proliferation in vivo. Immunofluorescent staining of control and BCL9-KD DCIS.COM and SUM225 MIND xenografts stained with the proliferation marker phospho-histone 3 (phosphoH3) (**a**) and an apoptotic marker cleaved caspase 3 (**b**) (*green*), and K5/K19 (*red*), and 4′,6-diamidino-2-phenylindole (DAPI). *White arrows* point to the cells with positive staining. The bar graphs represent the number of positive cells per 500 cells; the data represent the mean ± standard error of the mean (n = 4, *p <0.05)

To examine the role of BCL9 in invasive progression in vivo, BCL9 KD DCIS.COM and SUM225 cells, and control cells, were transplanted as MIND xenografts (Fig. 5a). Glands were collected at 10 weeks post-transplantation for DCIS.COM, and at 14 weeks for SUM225, and prepared for IF using antibodies for BCL9 to confirm in vivo KD, human cytokeratin 5 and 19 (K5 and K19) to detect in vivo growth of human DCIS-like lesions, SMA to detect the myoepithelial layer, phospho-histone 3 (phosphoH3) to detect cell proliferation, and cleaved caspase 3 to detect apoptosis. As shown (Fig. 5a), successful in vivo KD was achieved in both DCIS.COM and SUM225. The extent of invasion was analyzed by measuring the maximum distance traveled by the invasive cells past the myoepithelial layer of each mammary duct, and by counting the number of invasive lesions per gland (Fig. 5b). As shown in Fig. 5c, BCL9 KD DCIS.COM and SUM225 MIND lesions showed a significant reduction in the maximum distance of invasion (DCIS.COM = 78.0 ± 22.3 μm in KD compared to 302.7 ± 12.4 μm in control and for SUM225 = 75.5 ± 18.9 μm in KD compared to 338.3 ± 18.8 μm in control; *p* <0.05) and in the number of invasive lesions per field compared to the control (DCIS.COM = 1.4 ± 0.5 in KD compared to 4.3 ± 0.9 in control and for SUM225 = 3.0 ± 3.0 invasive lesions in KD compared to 11.7 ± 1.2 invasive lesions in control; *p* <0.05). As shown in Fig. 6a, BCL9 KD DCIS.COM and SUM225 showed a significant reduction in phosphoH3 compared to the control DCIS.COM and SUM225 (DCIS.COM = 3.25 ± 0.01 cells per 500 KD cells compared to 5.25 ± 0.25 cells per 500 control cells counted; SUM225 = 7.83 ± 0.01 cells per 500 KD cells compared to 21.5 ± 2.36 per 500 control cells counted; *p* <0.05). However, there was no change in the number of cleaved caspase 3-positive cells (Fig. 6b). These data demonstrate that BCL9 promotes in vivo cellular proliferation and invasion, while BCL9 is not involved in cell survival and viability.

### BCL9 regulates the expression of EMT biomarkers

**Fig. 7:**
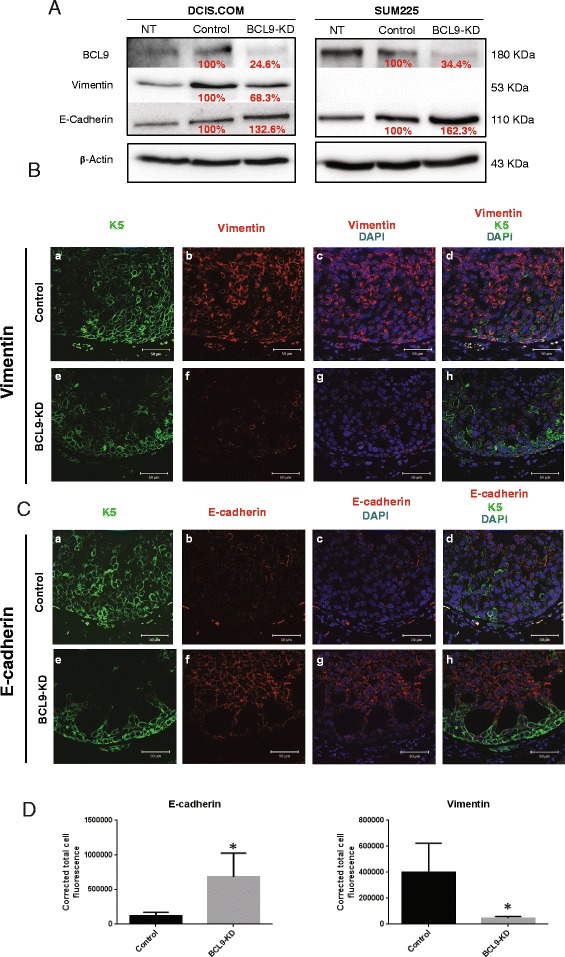
BCL9 KD reduced the mesenchymal markers and increased the luminal markers in DCIS.COM cell line. **a** Representative western blot analysis of cell lysates from non-transduced (NT), control and BCL9-KD DCIS.COM and SUM225 cells blotted with anti-BCL9, anti-vimentin, and anti-E-cadherin antibodies. β-actin was used as a loading control. The labels show percent change in BCL9-KD protein compared to control. **b** and **c** show immunofluorescent images of control (*a-d*) and BCL9-KD DCIS.COM (*e-h*) xenografts stained with vimentin (**b**) and E-cadherin (**c**) (*red*), and K5 (*green*), and 4′,6-diamidino-2-phenylindole (DAPI) in (*blue*). Scale bars =50 μm, × 40 magnification. **d** Bar graphs of cell fluorescence intensity units for E-cadherin and vimentin in control and BCL9 KD DCIS.COM cells. Measurements were obtained by ImageJ. Corrected cell fluorescence was calculated by subtracting the background mean density from the total integrated density. The data represent the mean ± standard error of the mean (n = 4, *p <0.05)

**Fig. 8:**
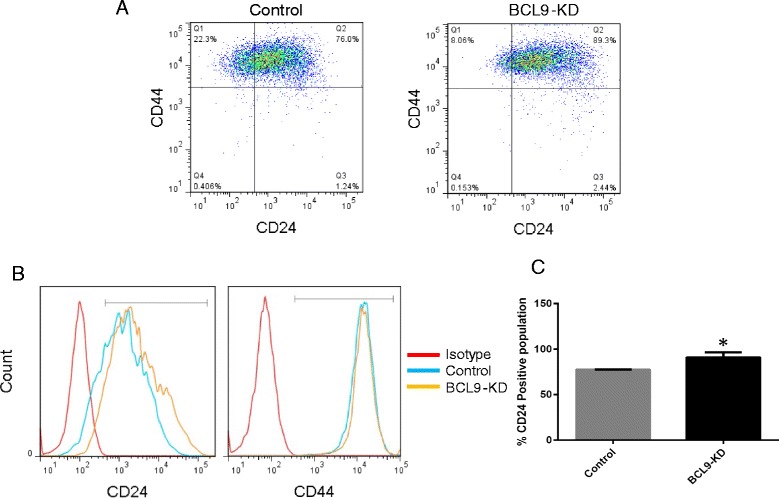
BCL9-KD increased CD24 positive population in DCIS.COM. **a** Representative flow analysis of control and BCL9-KD DCIS.COM cells for CD24 and CD44 (*top*), **b** representative histogram showing changes in CD24 (*left*) and CD44 (*right*) in control (*blue line*), BCL9-KD DCIS.COM cells (*yellow line*) compared to isotype control (*red line*). **c** Bar graphs show the percentage of CD24-positive population in BCL9 KD and control, the data represent the mean ± standard error of the mean

Previous studies in colon carcinoma and multiple myeloma models showed that tumors with BCL9 KD exhibited altered expression and distribution of mesenchymal and epithelial markers, vimentin, β-catenin and E-cadherin, indicative of reduced EMT [11]. Likewise, Deka, J and Colleagues [42] showed that mice with the conditionally deleted *Bcl9/Bcl9l* in intestinal cells exposed to a carcinogen (dimethylhydrazine followed by DSS) showed higher expression of both Wnt target genes that regulate EMT (vimentin, fibronectin and β-catenin) and stem-cell-related genes such as Sox6 compared to wild type. Based on these data, we proceeded with assessing the role of BCL9 on the expression of EMT biomarkers in our DCIS cell lines. Western blot was performed on cell lysates derived from DCIS.COM and SUM225 cells that were NT, expressed a scrambled shRNA control, or BCL9 KD using antibodies for vimentin as a mesenchymal marker and E-cadherin as an epithelial marker. BCL9 KD in DCIS.COM cells resulted in a reduction in vimentin and an increase in epithelial marker E-cadherin (Fig. 7a, Additional file 6: Figure S3A, and Additional file 8: Figure S5). SUM225 also showed an increase in E-cadherin, but control cells did not express vimentin, so as expected, BCL9 KD did not change vimentin expression. To confirm our findings in vivo, IF staining was performed on BCL9 KD DCIS.COM and SUM225 MIND xenografts, and their controls, using anti-vimentin, anti-E-cadherin, and anti-K5/K19 antibodies. Images were analyzed using ImageJ for fluorescence intensity. As shown in Fig. 7b-d, in our DCIS.COM MIND xenografts there was a significant increase in E-cadherin expression in BCL9 KD compared to the control (681,207 ± 198,349 U compared to 120,994 ± 25,258 U; *p* <0.05) and a significant reduction in vimentin expression in BCL9 KD compared to control (44,967 ± 5,402 U compared to 400,345 ± 111,633 U; *p* <0.05). DCIS.COM cells generate basal-like DCIS like lesions in vivo. However, in our SUM225 xenografts, there was not a significant reduction in vimentin and or upregulation in E-cadherin expression. SUM225 cells generate luminal-like DCIS lesions in vivo. These data suggest that BCL9 may have a more significant effect on the EMT-like phenotype in basal cells compared to luminal cells. The effects of BCL9 KD on the luminal marker CD24 and the basal marker CD44 in DCIS.COM and SUM225 were also assessed by flow cytometry analysis of NT, control and BCL9 KD cells. As seen in Fig. 8a-c (dot plot on the top panel and histogram on the lower panel), BCL9-KD cells showed an increase in the expression of luminal marker CD24 compared to the control in DCIS.COM (77.27 % ± 0.20 % vs 90.77 % ± 3.26 %; *p* <0.05), while there was no change in SUM225 cells (data not shown). CD44 expression levels did not change in either cell line, DCIS.COM or SUM225 (Fig. 8b; SUM225 data not shown). These data confirm our previous findings that BCL9 may contribute to the maintenance of an EMT program in some but not all cancer cell types.

### BCL9 interacts with β-catenin and enhances Wnt/β-catenin signaling

**Fig. 9:**
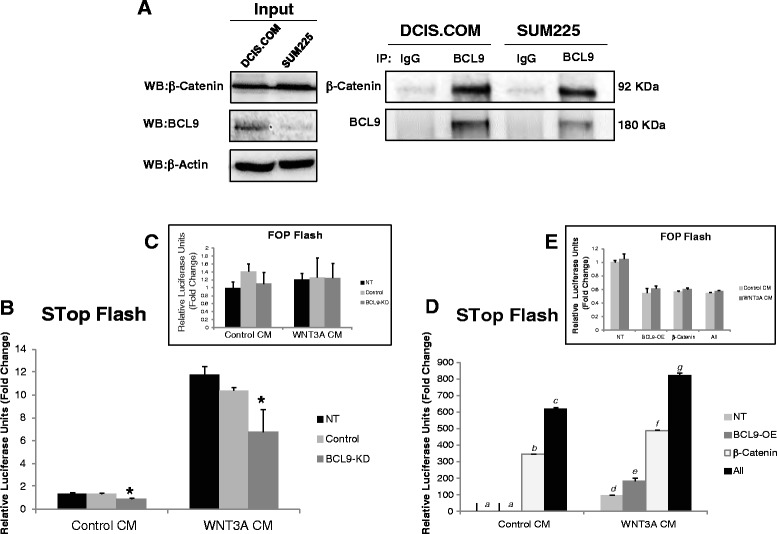
BCL9 interacted with β-catenin in DCIS cell lines and BCL9 KD inhibited the Wnt/β-catenin signaling activity. **a** Whole-cell extracts of DCIS.COM and SUM225 cells were immunoprecipitated for BCL9 using an anti-BCL9 antibody, followed by western blot analysis using anti-β-catenin and anti-BCL9 antibody. IgG antibody was used as a negative control for IP and 2 μg of whole cell lysate was used as input. **b** STopFlash reporter activity in DCIS.COM non-transduced (NT), control, and BCL9-KD, that were either treated with Wnt3A or control conditioned medium (CM). **c** FopFlash in these cells with control or Wnt3A CM. **d** STopFlash reporter activity in DCIS.COM NT, BCL9-OE, constitutively active β-catenin, or all combined that were either treated with Wnt3A or control CM. **e** FopFlash in these cells with control or Wnt3A CM. Data represent mean ± standard error of the mean (n = 3, * *p* <0.05, letters indicate statistically significant difference)

BCL9 and its homolog BCL9L and pygopus (PYGO) have been identified as co-activators for Wnt/ β-catenin transcription in *Drosophila* and in mammalian cells [11, 27]. To examine BCL9 and β-catenin interactions in our DCIS cell line models, BCL9 was immunoprecipitated from whole cell extracts of DCIS.COM and SUM225 cells using anti-BCL9 antibody, followed by western blot using anti β-catenin antibody. As shown in Fig. 9a, BCL9 interacts with β-catenin in both of our DCIS cell lines, DCIS.COM (Fig 9a, left panel) and SUM225 (Fig 9a, right panel). To explore whether BCL9 modulates Wnt/β-catenin-mediated transcription, we utilized the SuperTopFlash (STopflash), a luciferase reporter assay that measures β-catenin/LEF-TCF-mediated transcription, along with the FopFlash reporter with mutated LEF/TCF binding sites as a control. Non-transduced, control, and BCL9-KD DCIS.COM and SUM225 cells were transiently transfected along with STopFlash and FopFlash reporters, and treated with control or Wnt3A conditioned medium (CM) 4 h after transfection. Twenty-four hours after transfection, luciferase activity was measured. As shown in Fig. 9b and c, KD of BCL9 significantly reduced β-catenin/TCF-mediated transcription (*p* <0.05) in DCIS.COM, both in the presence and absence of Wnt3A stimulation, compared to similarly treated NT and controls, but not in SUM225 cells with or without Wnt3a stimulation (data not shown). In order to assess whether BCL9 enhances β-catenin mediated transcription, we overexpressed BCL9 and constitutively active β-catenin in human embryonic kidney (HEK) 293 T cells, which express low endogenous levels of BCL9 (data not shown). As expected, constitutively active β-catenin expression increased transcription, both in the absence and presence of Wnt3a stimulation, compared to non-transduced controls (NT; *p* <0.05; Fig. 9d). Overexpression of BCL9 (BCL9 OE) enhanced β-catenin/TCF-mediated transcription induced by Wnt3A by about two-fold compared to NT control (*p* <0.05). Furthermore, cells that overexpressed both BCL9 and constitutively active β-catenin showed significantly higher β-catenin/TCF-mediated transcription compared to β-catenin overexpression alone and in response to Wnt3A stimulation (approximately 1.7-fold increase; *p* <0.05). In addition, we analyzed canonical Wnt activation in BCL9 KD DCIS.COM cells after re-expression of BCL9 (BCL9-KD/OE). As shown in Additional file 7: Figure S4E, BCL9 KD/OE showed a significant increase in reporter activity compared to BCL9 KD with Wnt3a treatment (11.42 +/-0.46 vs. 3.3+/-0.76; p<0.05) and without wnt3a treatment (1.7+/-0.28 vs 0.6+/-0.13). These data demonstrate that BCL9, by binding to β-catenin, enhances canonical Wnt activation in the DCIS.COM cells (a basal cell line). While BCL9 still binds to β-catenin in SUM225 (luminal HER2 overexpressing) it does not activate Wnt canonical signaling that can be assessed by luciferase reporter.

### Enhanced BCL9 nuclear expression in DCIS with invasive component and high risk DCIS

**Fig. 10:**
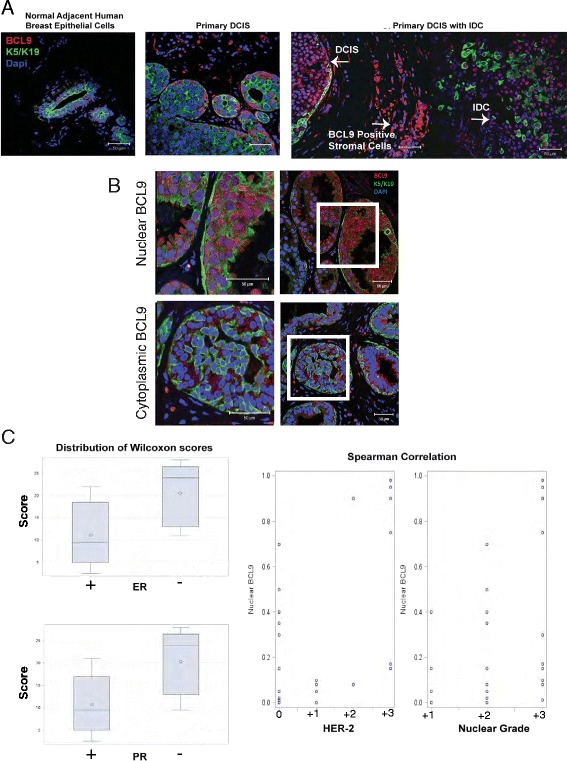
BCL9 may serve as a biomarker of high risk DCIS. **a** Immunofluorescence (IF) staining using anti-BCL9 antibody (*red*), anti-K5/K19 (*green*), and counterstained with 4′,6-diamidino-2-phenylindole (DAPI) (*blue*) in patient DCIS samples with and without invasion, and in normal adjacent human breast epithelial cells. **b** Representative IF images for patient DCIS samples with the above antibodies, demonstrating nuclear (*top*) and cytoplasmic (*bottom*) BCL9 expression patterns. **c** Statistical analysis of tissue sections from 28 patients purely with DCIS analyzed by IF using anti-BCL9 antibody. This analysis showed that the percentage of cells expressing nuclear BCL9 was significantly higher in DCIS that were estrogen receptor (ER)-negative (*p* = 0.004; Wilcoxon rank sum test), progesterone receptor (PR)-negative (*p* = 0.003; Wilcoxon rank-sum test), high human epidermal growth factor receptor 2(HER2) (Spearman correlation = 0.56; *p* = 0.002), and high nuclear grade (Spearman correlation = 0.49; *p* = 0.008). Median percentage (IQR) of cells positive for nuclear BCL9 for ER-positive samples = 8 % (1 %, 40 %); for PR-positive = 8 % (1 %, 35 %); ER-negative = 90 % (15 %, 95 %); PR-negative = 90 % (15 %, 95 %)

Recent strategies have demonstrated some utility in using expression of a limited gene set for predicting DCIS recurrence; however, the general use of this system is controversial [43]. Thus, finding biomarkers of DCIS high risk is still a research priority in breast cancer. To evaluate BCL9 as a potential biomarker for DCIS with high risk of recurrence, we initially examined the pattern of BCL9 expression using a tissue microarray (TMA) composed of samples from eight patients with DCIS (three patients with DCIS and IDC and five purely with DCIS). Figure 10a and b illustrate the three patterns of staining observed: weak cytoplasmic staining (adjacent normal; Fig. 10a left panel); mixed nuclear and cytoplasmic (Fig. 10a middle panel and Fig.10b lower panel); and enhanced nuclear expression (Fig. 10a right panel and Fig. 10b top panel). All adjacent normal breast epithelial cells expressed weak cytoplasmic BCL9 expression (similar to Fig. 10a left panel). Strikingly, all DCIS with IDC cases exhibited >90 % enhanced nuclear expression (similar to Fig. 10a right panel). Interestingly, enhanced BCL9 nuclear expression was associated with a loss of cytokeratin expression, which is indicative of EMT (as seen in Fig. 10a; right panel). We also observed increased expression of BCL9 in stromal macrophages (Fig. 10a; right panel). However, the role of BCL9 in stromal macrophages is beyond the scope of this study. Among the samples from patients purely with DCIS, comedo or cribriform DCIS exhibited enhanced BCL9 nuclear expression; those with papillary DCIS showed mixed nuclear and cytoplasmic BCL9 expression (data not shown).

As high nuclear BCL9 expression is present in DCIS with concurrent IDC, it may mean that this pattern predicts aggressive behavior; however, we do not know whether the patients with purely DCIS will have recurrence, nor do we know whether the lesions were completely excised, which could of course ensure a favorable outcome, even if the purely DCIS lesions with nuclear BCL9 were more aggressive. To begin to address this question, we examined the pattern of BCL9 expression in an expanded patient set that included 28 samples from patients who were diagnosed with DCIS. (Fig. 10c). In this set, we compared BCL9 localization with pathologic variables that are known to correlate with aggressive behavior and high risk for recurrence: nuclear grade, hormone receptor status and HER2 expression [44]. This analysis showed that DCIS lesions expressing higher numbers of nuclear BCL9-positive cells were more likely to be ER-negative (*p* = 0.004; Wilcoxon rank sum test), PR-negative (*p* = 0.003; Wilcoxon rank-sum test), high nuclear grade (Spearman correlation = 0.49; *p* = 0.008), and high HER2-expressing (Spearman correlation = 0.56; *p* = 0.002; Fig. 10c). Based on these data, BCL9 may serve as a future potential biomarker if validated in a larger dataset of DCIS patients with known outcome data.

Interestingly, analysis of TCGA data (provisional TCGA; 959 cases) [24] showed that 26 % of invasive breast cancers contain *BCL9* gene alterations. The majority of these alterations include amplification (13 %) and mRNA upregulation (17 %). This is a significant level of gene alteration when compared to *ESR1* (8 %), *ERBB2* (19 %) and BCL9L (5 %) (Additional file 9: Figure S6A). Furthermore, *BCL9* amplification is observed in a significantly higher proportion of invasive basal breast cancer (BLBC) subtypes compared to the other subtypes (Additional file 9: Figure S6B-C) [45–46]. Moreover, there is a significant association between *BCL9* gene amplification and mRNA upregulation (Additional file 9: Figure S6D). These data suggest that BCL9 may predispose to the development of basal-like invasive breast cancers. The TCGA data were also analyzed for the expression of differentially expressed genes in breast cancers that showed BCL9 upregulation. BCL9 upregulation was defined as BCL9 expression levels above the range defined by normal samples. An IPA analysis on the differentially expressed genes showed Wnt/β-catenin pathway to show a significant upregulation in BCL9-high compared to BCL9-low breast cancers (Additional file 10). A list of significant genes (1,756 downregulated and 980 upregulated) are listed in the Additional file 11.

## Discussion

Canonical Wnt signaling can be constitutively activated in cancer by a variety of mechanisms including mutations in adenomatous polyposis coli (APC), Axin, and β-catenin [47]. These mutations enable β-catenin to escape destruction and drive oncogenic Wnt signaling [47]. However, in breast cancer, where mutations in APC or β-catenin are not commonly reported, BCL9 overexpression may be a molecular mechanism contributing to aberrant Wnt activation and progression [11]. The mechanism by which BCL9 is overexpressed in some cancers is not entirely understood, but cancer genome analysis via GISTIC reveals copy number alterations in 13 % of all breast cancer cases examined [46]. BCL9 resides on chromosome 1q (1q21). Chromosome 1q amplification is a common finding in several cancers including breast [48]. BCL9 is a nuclear co-factor that, by binding to β-catenin and PYGO, modulates canonical Wnt signaling and promotes β-catenin-mediated transcription. The formation of a quaternary complex consisting of LEF/TCF, β-catenin, BCL9 and PYGO enhances β-catenin-dependent Wnt transcriptional activity [35]. Indeed, BCL9 is recognized as an adaptor that helps PYGO in recognizing modified histone H3 tails by their plant homeodomain (PHD) fingers [49]. The human *BCL9* and its paralog *BCL9L* reside on chromosome 1q21 and 11q23.3 respectively. Thus, the molecular regulation of these two genes may be very different. We did not find BCL9L upregulation to be associated with invasive progression in our DCIS MIND models. The role of BCL9L in breast cancer is currently unknown. One study reported BCL9L to regulate ER transcription by interaction with Sp1 through the proximal ESR1 gene promoter and to be highly expressed in patients with ER-positive breast cancers [37]. The exact role of BCL9 vs BCL9L in normal mammary gland development has not been studied.

We identified BCL9 by analysis of molecular profiling of DCIS at distinct stages of in situ to invasive transition. Our initial findings suggest that BCL9 expression and activity are important risk factors for breast cancer progression. This is based on the enhanced nuclear expression of BCL9 in DCIS epithelia that progress to invasion. Silencing of BCL9 in our invasive DCIS cell line led to in vivo and in vitro inhibition of both cell growth and invasion, and downregulation of vimentin, a biomarker of EMT. The role of BCL9 in the progression of other types of cancers has been reported previously. However, to our knowledge, there are currently no data on the role of BCL9 in breast cancer progression. Mani and colleagues [11] showed that KD of BCL9 by shRNA in a colon cancer cell line (colo320) and a multiple myeloma cell line (MM1S) caused a significant reduction in proliferation and colony formation. Overexpression of BCL9 increased colo320 and MM1S migration in transwell migration assays and in vitro Matrigel-coated invasion assays. Immunocompromised mice injected with colo320 KD of BCL9 showed significant increase in survival and reduced lung metastasis. Likewise, mice injected with MM1S cells KD BCL9 also showed improved survival and reduced metastasis to the long bones, spine, and head. BCL9 KD tumors also showed reduced EMT markers such as vimentin, E-cadherin and β-catenin.

We demonstrated that BCL9 KD resulted in suppression of Wnt signaling as assessed by TOP-FLASH Wnt reporter assays in our basal DCIS cell line (DCIS.COM), while BCL9 KD in SUM225 (luminal HER2 overexpressing) did not affect the canonical Wnt signaling. This result may indicate that the canonical Wnt pathway is involved in the progression of certain subtypes of breast cancer i.e., basal subtypes. This observation is interesting because the TCGA breast cancer data shows that BCL9 is significantly amplified in basal subtypes of breast cancers [46, 50]. However, there is also the possibility that TopFlash used in our study did not detect Wnt activity because this reporter does not detect all transcriptional effects in Wnt signaling.

Our studies also suggest that BCL9 may serve as a future biomarker of high-risk DCIS if validated in a large dataset of DCIS patients with known outcome. By analysis of 28 DCIS patient samples, we demonstrated that DCIS lesions expressing higher nuclear BCL9 (percentage of cells expressing nuclear BCL9) were more likely to be ER-negative, PR-negative, high nuclear grade, and high in HER2 expression. These characteristics are associated with higher recurrence rate in DCIS [44].

## Conclusion

Collectively, the findings in this study suggest that BCL9, by enhancement of canonical Wnt signaling and initiating an EMT program, serves as an important molecular driver in invasive transition of human DCIS. Therefore, BCL9 may serve as a potential future biomarker of high-risk DCIS and as a therapeutic target for prevention of IDC.

## Additional files

Additional file 1: Table S1. List of antibodies and sources for use in immunofluorescent staining, co-immunoprecipitation, western analysis, and FACS analysis. (PDF 245 kb)
Additional file 2: Figure S1. Majority of SUM225 and DCIS.COM cells were epithelial cell adhesion molecule (*EPCAM*)-positive by flow analysis. Representative flow analysis of (**A**) SUM225 (*left*) and DCIS.COM cells (*right*) for EpCAM (*black line*) compared to isotype control (*gray line*) showed that 95 % of SUM225 cells and 99 % of DCIS.COM cells were EPCAM-positive. **B** Histogram overlaying SUM225 and DCIS.COM EPCAM positive cells. **C** Flow analysis for EPCAM positive cells in BCL9 KD (*gray line*) and control (*black line*) SUM225 cells at 6 weeks post intraductal injection. **D** Bar graphs representing EPCAM expression levels in BCL9 KD and Control cells show no statistically significant differences among the groups (n = 3). (PDF 417 kb)
Additional file 3:
**Differentially expressed genes in DCIS.COM and SUM225 mouse intraductal xenograft model (MIND) xenografts during transition from 2 to 6 weeks.** Significance analysis for microarrays (SAM) software was utilized to determine differentially expressed genes between the 2- to 6-week time point in both DCIS.COM and SUM225 cell lines. The cutoff for significance was determined by <5 % false discovery rate. Two-class unpaired SAM analysis generated a list of significant genes and fold-change values between 2 and 6 weeks in DCIS.COM (18,590 downregulated; 10,227 upregulated) and SUM225 (19,953 downregulated and 14,691 upregulated). (XLSX 3297 kb)
Additional file 4:
**Wnt-pathway-specific genes differentially expressed in ductal carcinoma in situ DCIS.COM and SUM225 mouse intraductal xenograft model (MIND) xenografts during transition from 2 to 6 weeks.** Genes significantly differentially expressed between the 2- to 6-week time point in both DCIS.COM and SUM225 cell lines were further analyzed using Ingenuity Pathway Analysis® (IPA). The Wnt/β-catenin canonical pathway was identified as a significantly upregulated pathway in both cell line MIND xenografts during transition from 2 to 6 weeks. (XLS 48 kb)
Additional file 5: Figure S2. BCL9 showed increased nuclear expression, while BCL9L expression remained cytoplasmic during ductal carcinoma in situ (*DCIS*) invasive transition. **A** Immunofluorescence staining of BCL9 (*red*; *top panel*), BCL9L (*red*; *bottom panel*), K5/K19 (*green*), and Hoechst (*blue*) in a primary sample that represents: adjacent normal glands (*left*), DCIS lesions (*middle*), and invasive ductal carcinoma (*IDC*) (*right*). BCL9 and BCL9L are conjugated to Alexa-Fluor 594 (*red*) and K5/K19 were conjugated to Alexa-Fluor 488 (*green*). Nuclei were counterstained with Hoechst. Scale bars 50 μm, × 40 objective was used. **B**, **C** RT-qPCR of BCL9 and BCL9L mRNA in epithelial cell adhesion molecule (*EpCAM*)-positive epithelial cells sorted from SUM225 (**B**) and DCIS.COM (**C**) mouse intraductal xenograft model (*MIND*) xenografts at 2, 6, and 10 weeks post-intraductal injection. The bar graphs represent fold change normalized to 2 weeks. Data are mean values ± standard error of the mean (n = 3, **p* <0.05). **D** Representative western blot analysis of cell lysates from control and BCL9-KD-SUM225 and DCIS.COM blotted with anti-BCL9, and anti-BCL9L antibodies. β-actin was used as a loading control. The analysis showed no change in BCL9L protein levels in BCL9-KD cells compared to control cells. (PDF 5012 kb)
Additional file 6: Figure S3. MTS, migration and invasion assays in ductal carcinoma in situ (*DCIS*) BCL9-KD cells with shRNA 2. **A** Western blot analysis using anti-BCL9, anti-vimentin, anti-E-cadherin antibodies, and anti-β-actin antibody as a loading control. **B** MTS assays of scrambled control 2 (*control 2*) and BCL9-KD 2 in DCIS.COM (*left bar graph*) and SUM225 (*right bar graph*). Bar graphs represent mean absorbance at 490 nm normalized to control ± standard error of the mean (SEM) (n = 3, **P* <0.05). **C**, **D** Representative images of the migration and invasion assays. Bar graph represent percent area of cells migrated (*left*) and invaded (*right*) under the membrane after 24 h for DCIS.com and 96 h for SUM225. Invasion and migration were determined by ImageJ analysis of microscopic images per sample, the data are mean values normalized to control ± SEM (n = 3, **P* <0.05). **E** STopFlash and FopFlash reporter activity in DCIS.COM control 2, and BCL9-KD 2, which were either treated with Wnt3A or control conditioned medium (CM). Data represent mean ± SEM (n = 3, **P* <0.05). (PDF 650 kb)
Additional file 7: Figure S4. MTS, migration and invasion assays in *DCIS*.COM cells that were previously transduced with scrambled control (*Control*) or BCL9 KD shRNA. The control cells and BCL9 KD cells were re-transduced with empty vector (*EV*), BCL9 overexpression (*BCL9-OE*) and BCL9 KD. BCL9-OE was achieved by transduction using the PCDH-BCL9 (BCL9-OE) acquired from Dr. Carrasco [11]. **A** Western blot analysis was performed using anti-BCL9, anti-vimentin, anti-E-cadherin antibodies, and anti-β-actin as a loading control. **B** MTS assay on control cells transduced with EV (*control + EV*), or BCL9-OE (*control + BCL9-OE*), BCL9-KD transduced with EV (*BCL9 KD + EV*), and BCL9-KD transduced with BCL9-OE (*BCL9 KD + BCL9-OE*). Bar graphs represent mean absorbance at 490 nm normalized to control ± standard error of the mean (SEM) (n = 6). **C**, **D** Representative images of the migration and invasion assays. Bar graph represents percent area of cells migrated (*left*) and invaded (*right*) under the membrane after 24 h. Invasion and migration were determined by ImageJ analysis of microscopic images per sample, the data are mean values normalized to control ± SEM (n = 3). **E** TopFlash and FopFlash reporter activity in DCIS.COM transduced as above that were either treated with Wnt3A or control conditioned medium (CM). Data represent mean ± SEM (n = 3, *letters* indicate statistically significant difference). (PDF 964 kb)
Additional file 8: Figure S5. Bar graphs represent densitometry of BCL9, E-cadherin, and vimentin in non-transduced (NT), control, and BCL9 KD *DCIS*.COM (**A**) (n = 4), and BCL9 and E-cadherin in NT, control, and BCL9 KD SUM225 (**B**) (n = 3). Data represent mean ± standard error of the mean (**P* <0.05). (PDF 492 kb)
Additional file 9: Figure S6. A significant proportion of breast cancers showed *BCL9* gene alteration. **A** The Cancer Genome Atlas (TCGA) provisional data showed that 26 % of invasive breast cancers (total of 965 cases) contained a genetic alteration in the *BCL9* gene; the majority of these consisted of gene amplification and mRNA upregulation. This was a significant level of genetic alteration when compared to *ERBB2* (19 %), *ESR1* (8 %) and *BCL9L* (5 %). **B** *BCL9* gene alterations across all cancers. The *BCL9* gene was altered in many cancers including breast, liver and bladder. The arrow points to invasive breast cancers showing 14 % gene alteration (135 in 962 cases). **C**, **D** TCGA data showed that a significantly higher proportion of basal breast cancers contained *BCL9* genomic amplification compared to the other subtypes (total of 825 cases). **E** The gene expression data were available as *z* scores. Diploid classification was used to identify median gene expression value, and this value was used as the cutoff for dichotomizing gene expression as low (<= median) or high (>median) for BCL9 expression. Contingency tables were created for diploid vs amplified or gain vs amplified against low or high BCL9 expression. Chi-square analysis indicated significant association between BCL9 amplification and high levels of BCL9 gene expression compared to diploid samples. (PDF 3138 kb)
Additional file 10:
**Analysis of The Cancer Genome Atlas (TCGA) data showing canonical Wnt pathways genes expressed in BCL9-high versus BCL9-low tumors.** TCGA breast cancer samples with BCL9 levels above the range defined by normal samples were labeled upregulated in cancer (414 samples). The significant differentially expressed genes from TCGA analysis of BCL9-high versus BCL9-low tumors were analyzed in Ingenuity Pathway Analysis (IPA) and the canonical pathways with a *p* value ≤0.05 were obtained. (XLS 124 kb)
Additional file 11:
**Analysis of the The Cancer Genome Atlas (TCGA) data showed differentially expressed genes in BCL9-high versus BCl9-low tumors.** Differential gene expression was performed between TCGA breast cancer samples with a normal range of BCL9 and samples with upregulated BCL9. A list of significant genes (1,756 down regulated 980 upregulated) was obtained with a threshold of a false discovery rate ≥0.05 and log fold change 0.26. (XLSX 1492 kb)

## Abbreviations

aCGH: array comparative genomic hybridization
APC: adenomatous polyposis coli
BCL9: B cell lymphoma-9
BCL9L: B cell lymphoma-9-like
BLBC: basal-like breast cancer
bp: base pairs
CD24: cluster of differentiation 24
CD44: cluster of differentiation 24
CM: conditional medium
DAPI: 4′,6-diamidino-2-phenylindole
DCIS: ductal carcinoma in situ
DMEM: Dulbecco’s modified Eagle’s medium
EMT: epithelial mesenchymal transition
EpCAM: epithelial cell adhesion molecule
Erbb2: FDR, false discovery rate
Esr1: ER, estrogen receptor (gene and protein)
H&E: hematoxylin and eosin
HER2: human epidermal growth factor receptor 2 (gene and protein)
IDC: invasive ductal carcinoma
IF: immunofluorescence
IPA: Ingenuity Pathway Analysis
K19: keratin 19
K5: keratin 5
LEF1: lymphoid enhancer-binding factor 1
MIND: mouse intraductal xenograft model
NSG: NOD-SCID IL2Rgamma null
NT: non-transduced
PBS: phosphate-buffered saline
PR: progesterone receptor
PYGO: Pygopus
qPCR: quantitative PCR
SAM: significance analysis of microarrays
SEM: standard error of the mean
SMA: smooth muscle actin
SOX6: SRY (sex determining region Y)-Box 6
SP1: specificity protein 1
TCF: T cell specific, HMG-box
TCGA: The Cancer Genome Atlas
TGFβ: transforming growth factor beta
TMA: tissue microarray
