# Supplementary material for: Expression profiling of in vivo ductal carcinoma in situ progression models identified B cell lymphoma-9 as a molecular driver of breast cancer invasion
Source: Breast Cancer Res. 2015 Sep 17;17:128. doi: 10.1186/s13058-015-0630-z (PMC4574212; doi:10.1186/s13058-015-0630-z)
Supplement: Additional file 1: Table S1. — List of antibodies and sources for use in immunofluorescent staining, co-immunoprecipitation, western analysis, and FACS analysis. (PDF 245 kb) [file 13058_2015_630_MOESM1_ESM.pdf]

	Company	Catalog #	Isotype
**IF staining**			
Primary antibodies			
BCL9	Abcam	37305	Rabbit
BCL9L	Abcam	113110	Rabbit
Cytokeratin 5	Vector	VPC400	Mouse
Cytokeratin 19	Thermoscientific	MS198	Mouse
SMA	Thermoscientific	PA5-18292	Goat
Phospho-histone3	Abcam	ab47297	Rabbit
Cleaved caspase 3	Cell signaling	9664S	Rabbit
Secondary antibodies			
Alexa Fluor 488 anti-rabbit	Invitrogen	A11008	Goat
Alexa Fluor 594 anti-mouse	Invitrogen	A21203	Donkey
Alexa Fluor 488 anti-mouse	Invitrogen	A11012	Goat
**Co-immunoprecipitation**			
IgG	Cell Signaling	2729S	Rabbit
BCL9	Santa Cruz	sc-68915	Rabbit
**Western blot analysis**			
BCL9	Abcam	37305	Rabbit
β-catenin	BD	610153	Mouse
β-actin	Chemicon	MAB1501	Mouse
BCL9L	Abcam	113110	Rabbit
**FACS analysis**			
Human CD44 (PE)-conjugated	BD Pharmingen	555479	
Human CD24 (FITC)-conjugated!	BD Pharmingen	555427	
