# Supplementary material for: Expression profiling of in vivo ductal carcinoma in situ progression models identified B cell lymphoma-9 as a molecular driver of breast cancer invasion
Source: Breast Cancer Res. 2015 Sep 17;17:128. doi: 10.1186/s13058-015-0630-z (PMC4574212; doi:10.1186/s13058-015-0630-z)
Supplement: Additional file 2: Figure S1. — Majority of SUM225 and DCIS.COM cells were epithelial cell adhesion molecule (EPCAM)-positive by flow analysis. Representative flow analysis of (A) SUM225 (left) and DCIS.COM cells (right) for EpCAM (black line) compared to isotype control (gray line) showed that 95 % of SUM225 cells and 99 % of DCIS.COM cells were EPCAM-positive. B Histogram overlaying SUM225 and DCIS.COM EPCAM positive cells. C Flow analysis for EPCAM positive cells in BCL9 KD (gray line) and control (black line) SUM225 cells at 6 weeks post intraductal injection. D Bar graphs representing EPCAM expression levels in BCL9 KD and Control cells show no statistically significant differences among the groups (n = 3). (PDF 417 kb) [file 13058_2015_630_MOESM2_ESM.pdf]

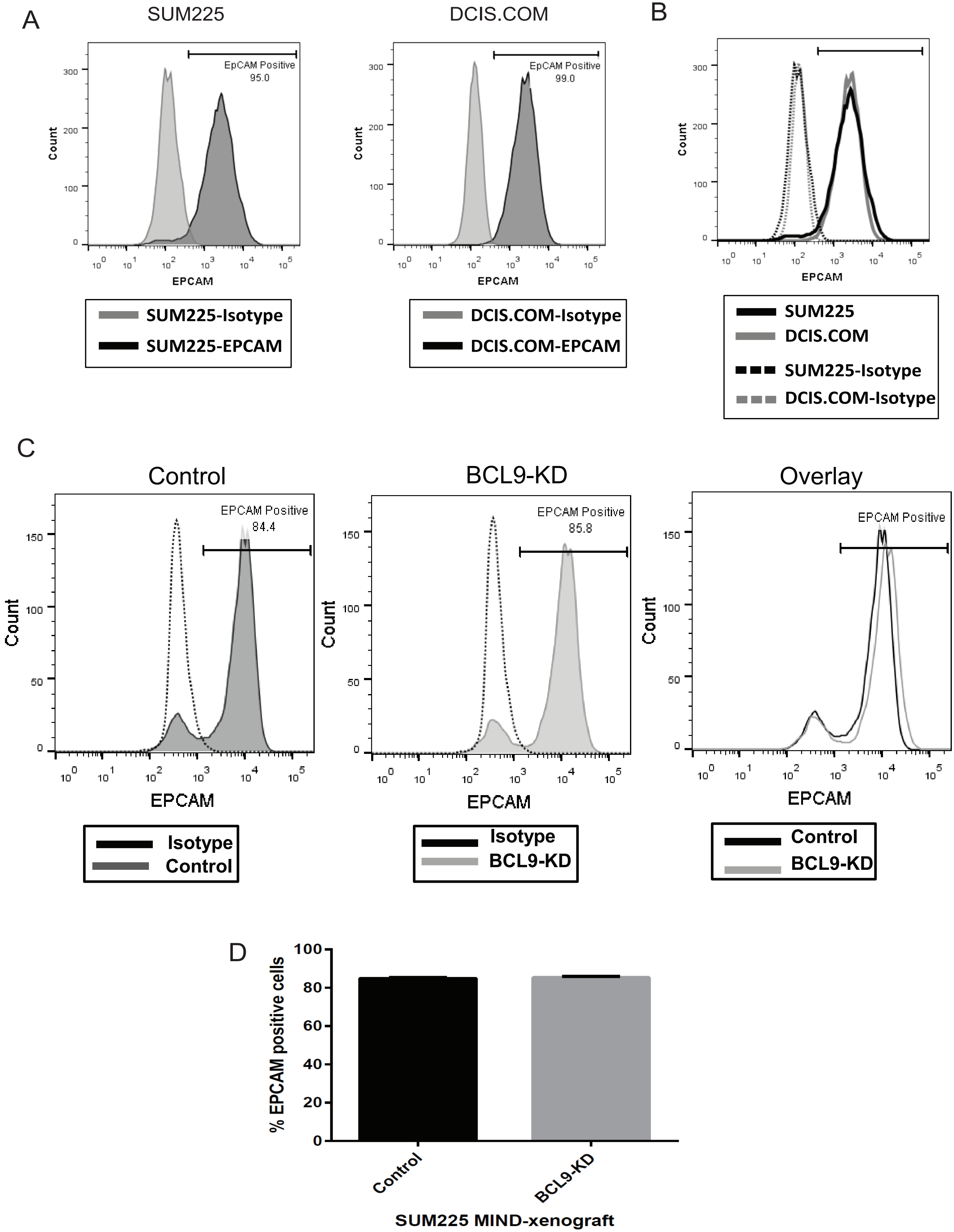
A
SUM225
DCIS.COM
EpCAM Positive
95.0
EpCAM Positive
99.0
Count
EPCAM
SUM225-Isotype
SUM225-EPCAM
DCIS.COM-Isotype
DCIS.COM-EPCAM
B
Count
EPCAM
SUM225
DCIS.COM
SUM225-Isotype
DCIS.COM-Isotype
C
Control
BCL9-KD
Overlay
EPCAM Positive
84.4
EPCAM Positive
85.8
EPCAM Positive
Count
EPCAM
Isotype
Control
Isotype
BCL9-KD
Control
BCL9-KD
D
% EPCAM positive cells
Control
BCL9-KD
SUM225 MIND-xenograft
