# Supplementary figures and images for: Expression profiling of in vivo ductal carcinoma in situ progression models identified B cell lymphoma-9 as a molecular driver of breast cancer invasion
Source: Breast Cancer Res. 2015 Sep 17;17:128. doi: 10.1186/s13058-015-0630-z (PMC4574212; doi:10.1186/s13058-015-0630-z)

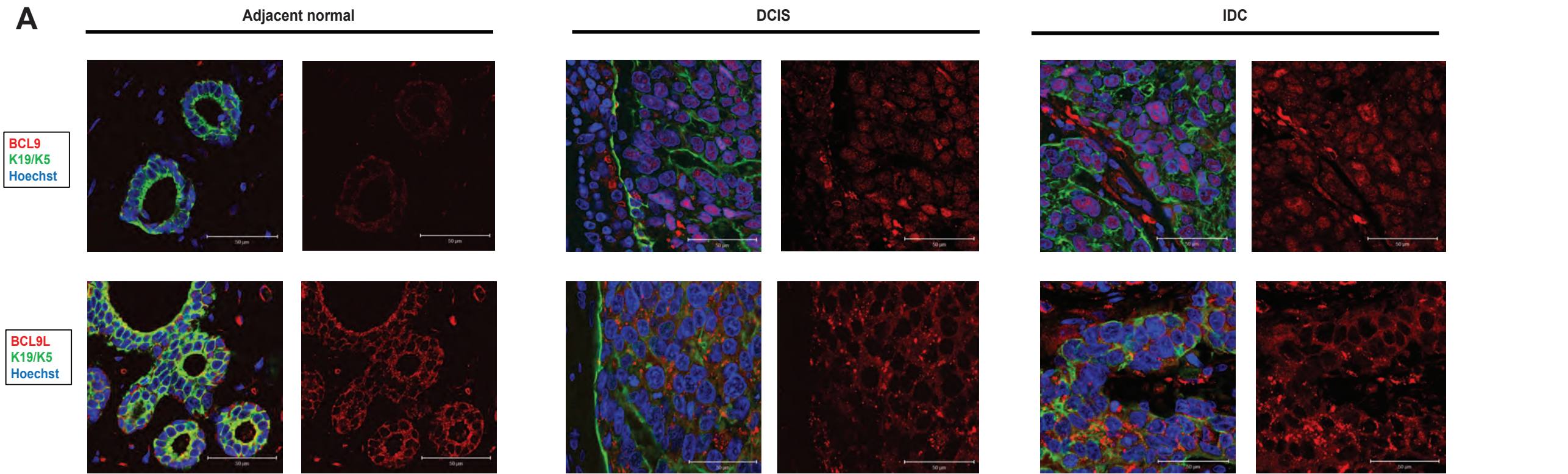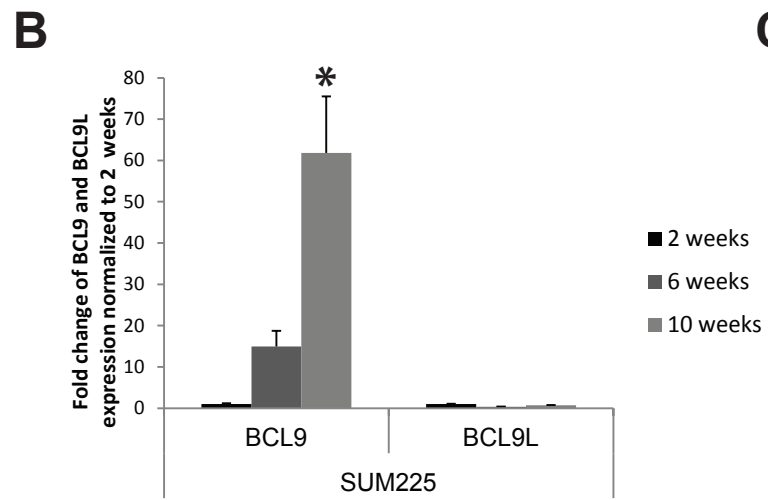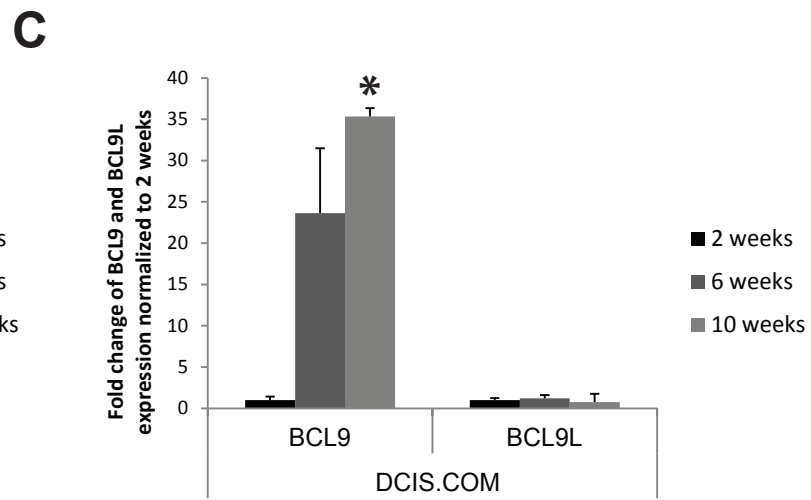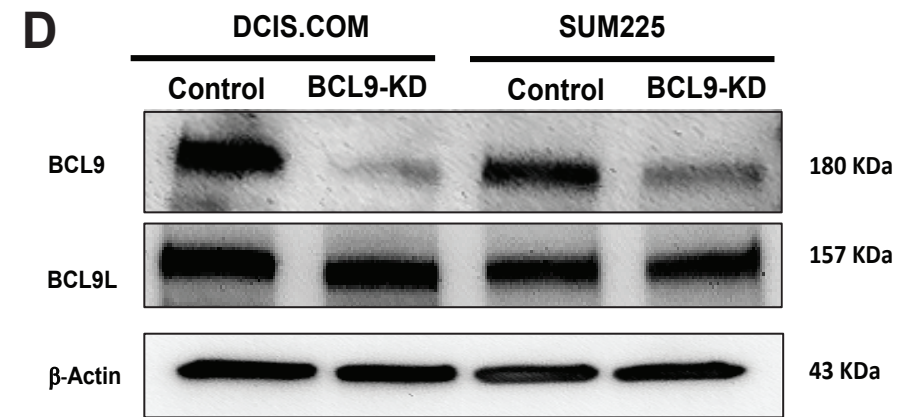

Supplement: Additional file 5: Figure S2. — BCL9 showed increased nuclear expression, while BCL9L expression remained cytoplasmic during ductal carcinoma in situ (DCIS) invasive transition. A Immunofluorescence staining of BCL9 (red; top panel), BCL9L (red; bottom panel), K5/K19 (green), and Hoechst (blue) in a primary sample that represents: adjacent normal glands (left), DCIS lesions (middle), and invasive ductal carcinoma (IDC) (right). BCL9 and BCL9L are conjugated to Alexa-Fluor 594 (red) and K5/K19 were conjugated to Alexa-Fluor 488 (green). Nuclei were counterstained with Hoechst. Scale bars 50 μm, × 40 objective was used. B, C RT-qPCR of BCL9 and BCL9L mRNA in epithelial cell adhesion molecule (EpCAM)-positive epithelial cells sorted from SUM225 (B) and DCIS.COM (C) mouse intraductal xenograft model (MIND) xenografts at 2, 6, and 10 weeks post-intraductal injection. The bar graphs represent fold change normalized to 2 weeks. Data are mean values ± standard error of the mean (n = 3, *p <0.05). D Representative western blot analysis of cell lysates from control and BCL9-KD-SUM225 and DCIS.COM blotted with anti-BCL9, and anti-BCL9L antibodies. β-actin was used as a loading control. The analysis showed no change in BCL9L protein levels in BCL9-KD cells compared to control cells. (PDF 5012 kb) [file 13058_2015_630_MOESM5_ESM.pdf]

A

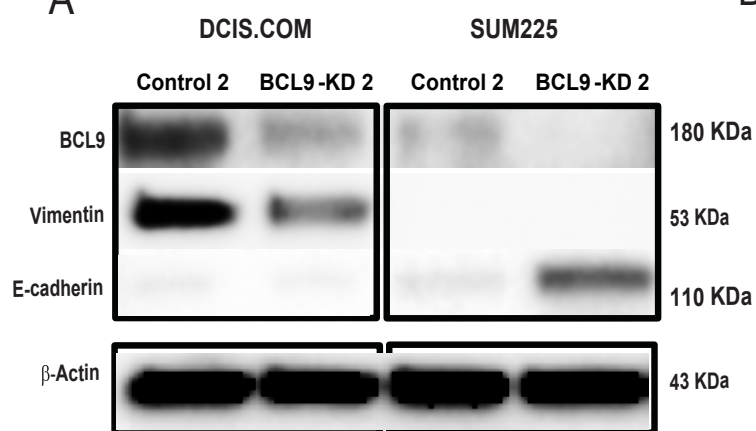

B

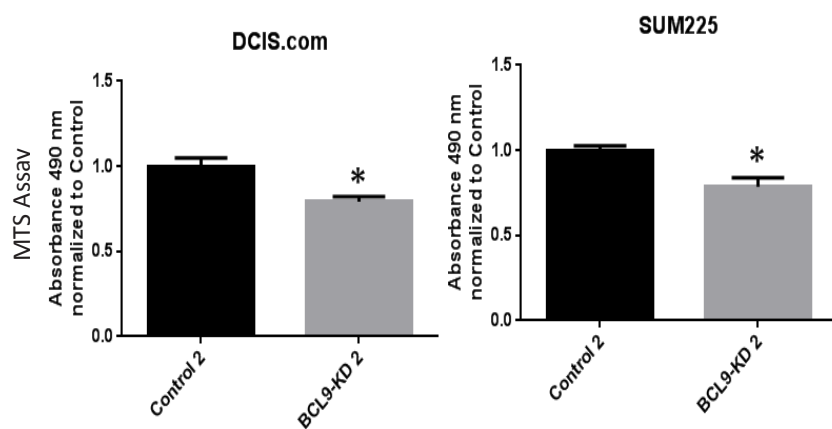

C

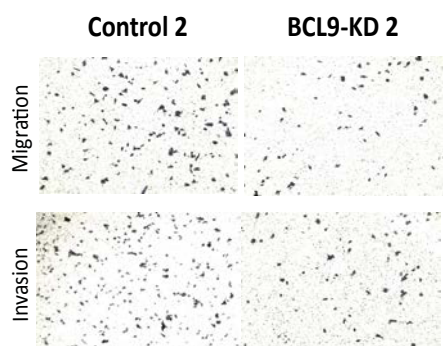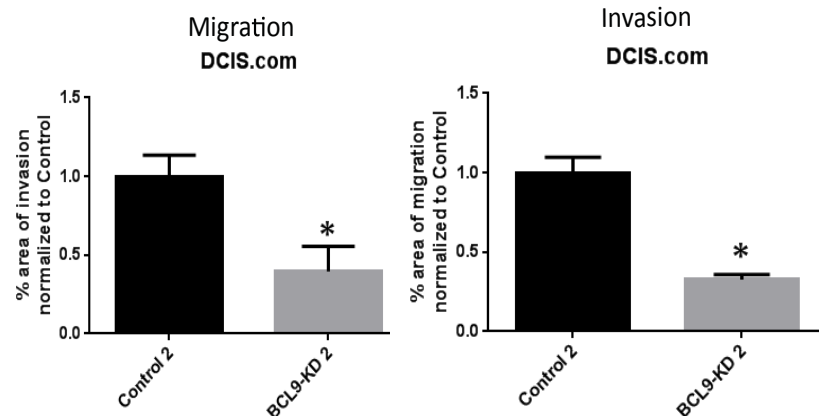

D

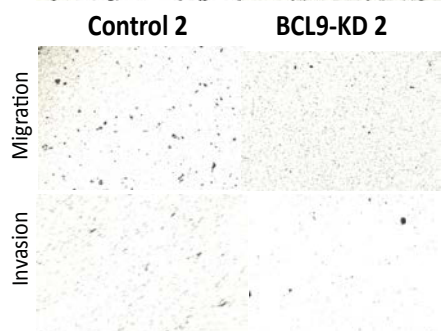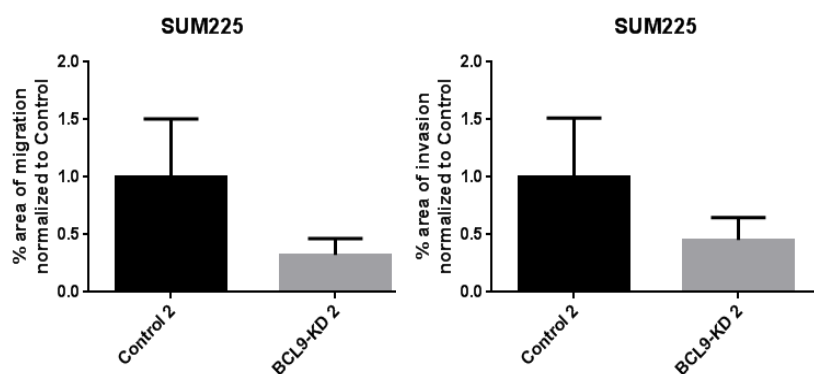

E

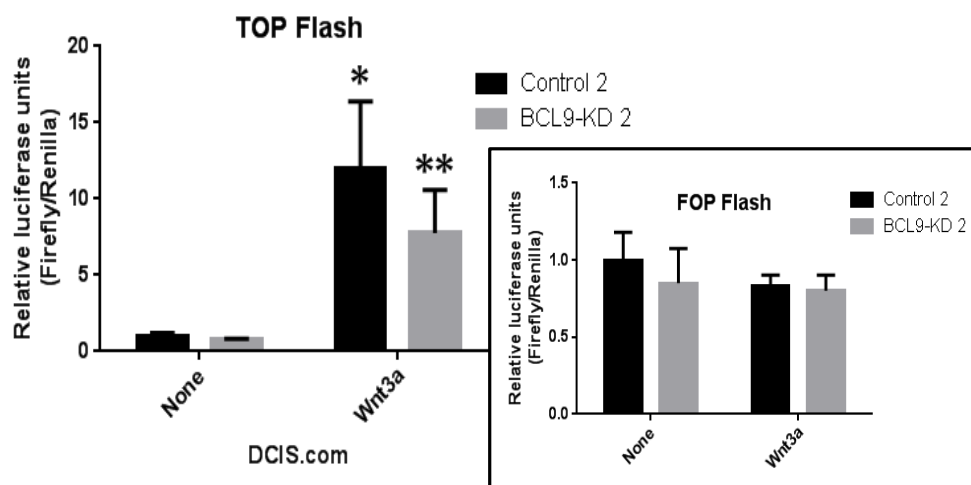

Supplement: Additional file 6: Figure S3. — MTS, migration and invasion assays in ductal carcinoma in situ (DCIS) BCL9-KD cells with shRNA 2. A Western blot analysis using anti-BCL9, anti-vimentin, anti-E-cadherin antibodies, and anti-β-actin antibody as a loading control. B MTS assays of scrambled control 2 (control 2) and BCL9-KD 2 in DCIS.COM (left bar graph) and SUM225 (right bar graph). Bar graphs represent mean absorbance at 490 nm normalized to control ± standard error of the mean (SEM) (n = 3, *P <0.05). C, D Representative images of the migration and invasion assays. Bar graph represent percent area of cells migrated (left) and invaded (right) under the membrane after 24 h for DCIS.com and 96 h for SUM225. Invasion and migration were determined by ImageJ analysis of microscopic images per sample, the data are mean values normalized to control ± SEM (n = 3, *P <0.05). E STopFlash and FopFlash reporter activity in DCIS.COM control 2, and BCL9-KD 2, which were either treated with Wnt3A or control conditioned medium (CM). Data represent mean ± SEM (n = 3, *P <0.05). (PDF 650 kb) [file 13058_2015_630_MOESM6_ESM.pdf]

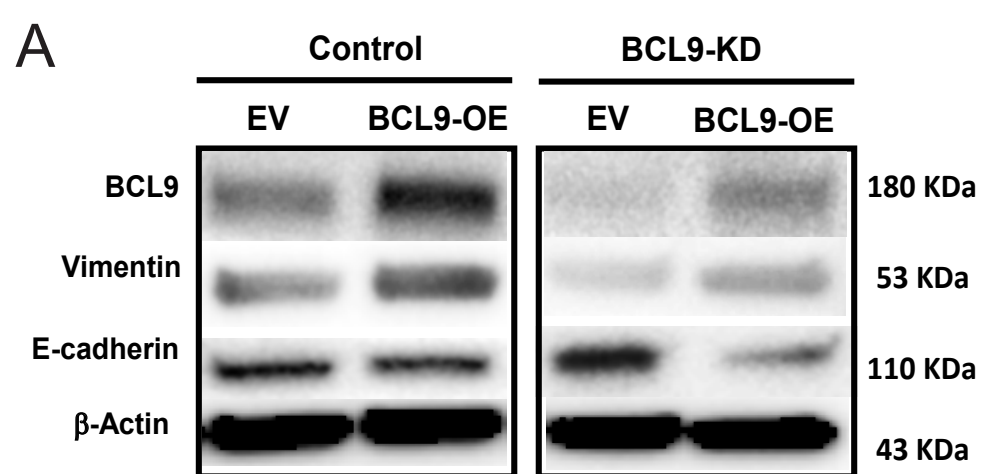

**B**

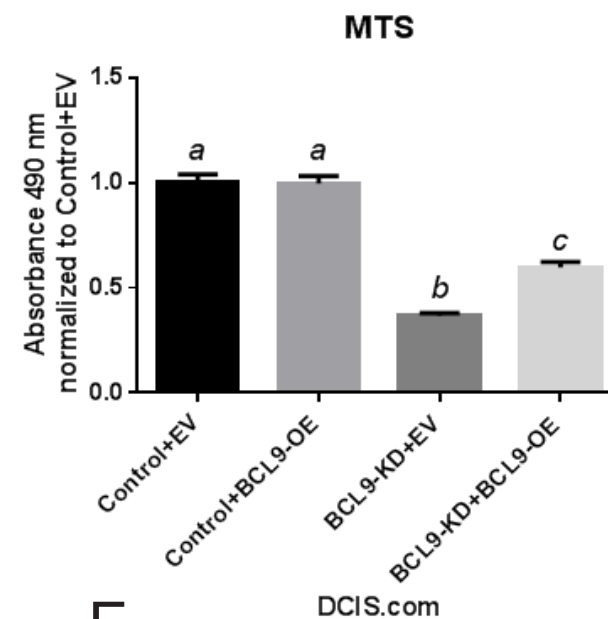

**C**

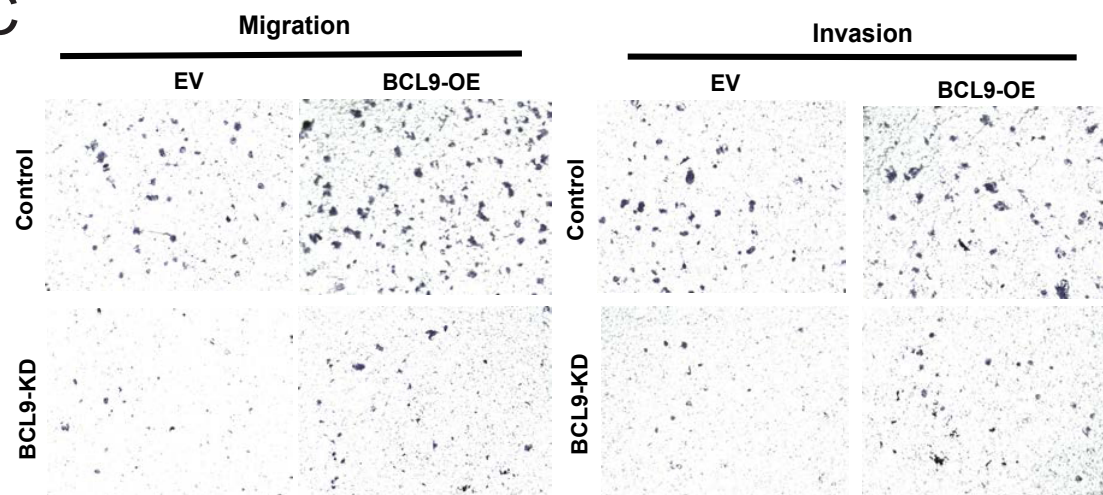

**D**

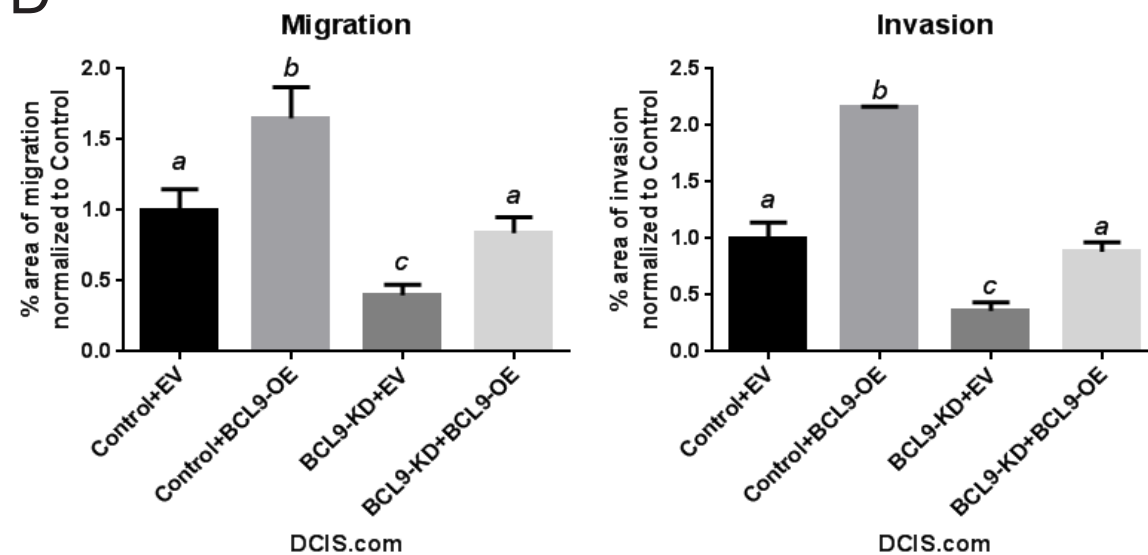

**E**

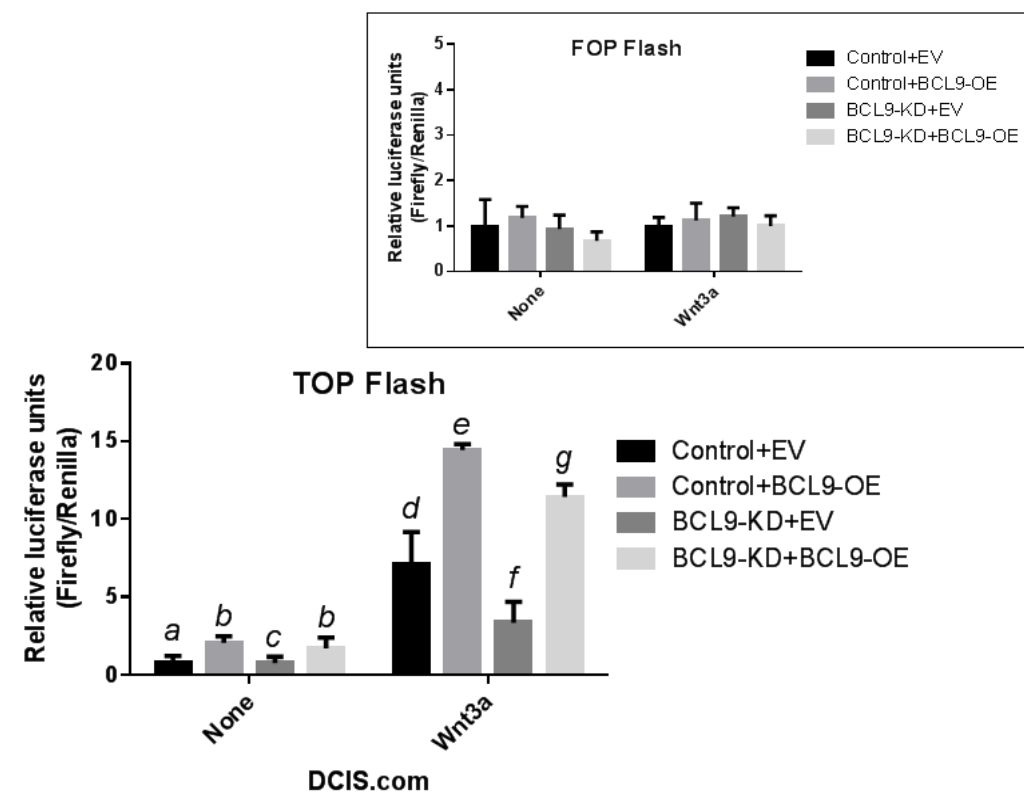

Supplement: Additional file 7: Figure S4. — MTS, migration and invasion assays in DCIS.COM cells that were previously transduced with scrambled control (Control) or BCL9 KD shRNA. The control cells and BCL9 KD cells were re-transduced with empty vector (EV), BCL9 overexpression (BCL9-OE) and BCL9 KD. BCL9-OE was achieved by transduction using the PCDH-BCL9 (BCL9-OE) acquired from Dr. Carrasco [11]. A Western blot analysis was performed using anti-BCL9, anti-vimentin, anti-E-cadherin antibodies, and anti-β-actin as a loading control. B MTS assay on control cells transduced with EV (control + EV), or BCL9-OE (control + BCL9-OE), BCL9-KD transduced with EV (BCL9 KD + EV), and BCL9-KD transduced with BCL9-OE (BCL9 KD + BCL9-OE). Bar graphs represent mean absorbance at 490 nm normalized to control ± standard error of the mean (SEM) (n = 6). C, D Representative images of the migration and invasion assays. Bar graph represents percent area of cells migrated (left) and invaded (right) under the membrane after 24 h. Invasion and migration were determined by ImageJ analysis of microscopic images per sample, the data are mean values normalized to control ± SEM (n = 3). E TopFlash and FopFlash reporter activity in DCIS.COM transduced as above that were either treated with Wnt3A or control conditioned medium (CM). Data represent mean ± SEM (n = 3, letters indicate statistically significant difference). (PDF 964 kb) [file 13058_2015_630_MOESM7_ESM.pdf]

**A**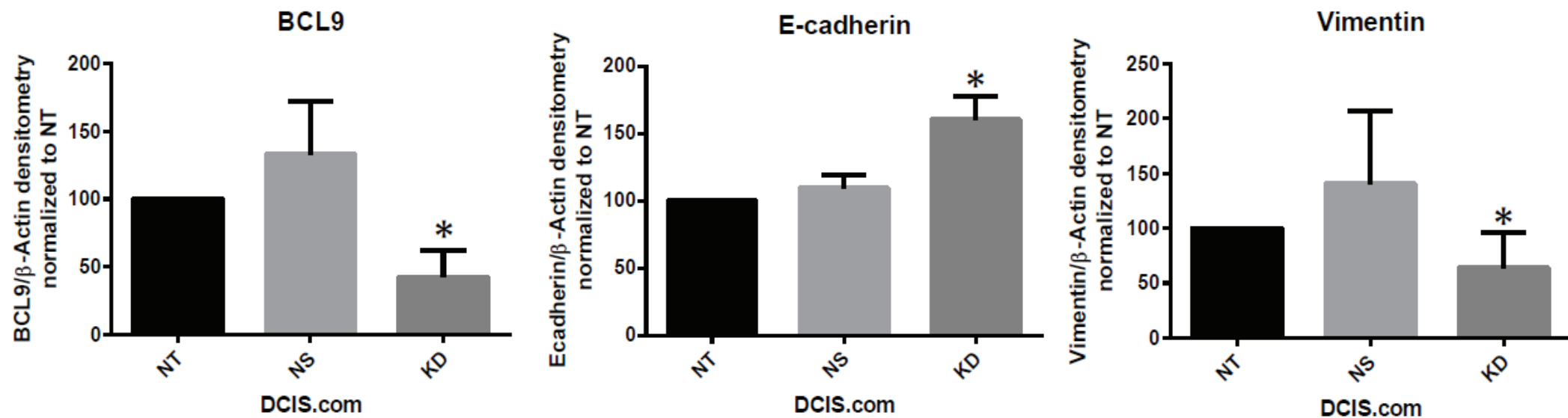**B**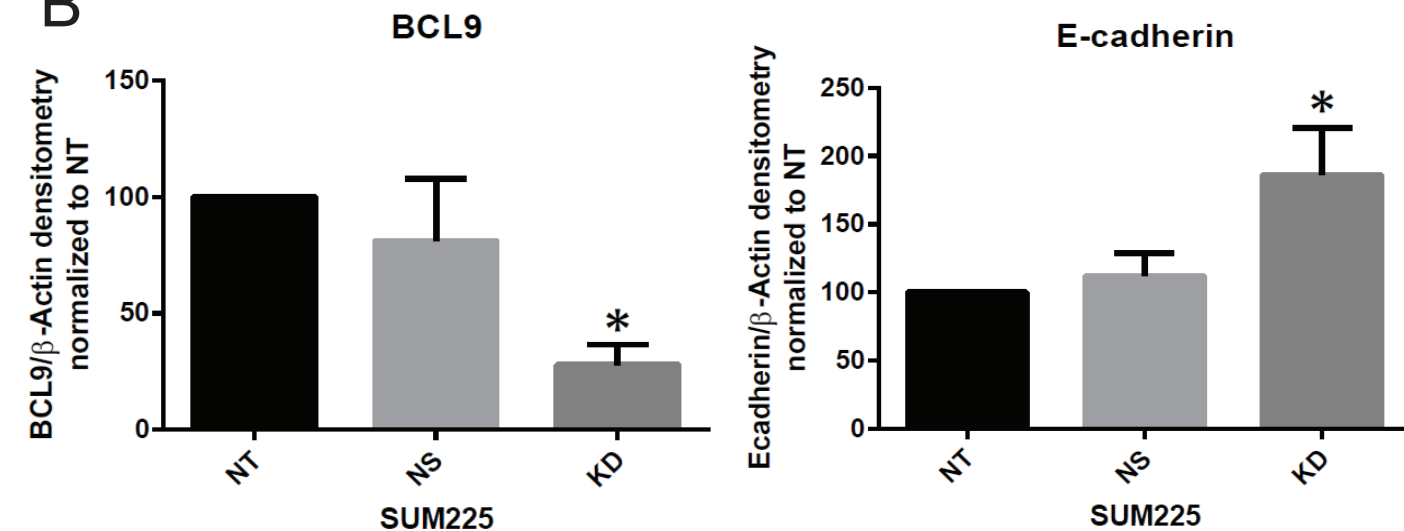

Supplement: Additional file 8: Figure S5. — Bar graphs represent densitometry of BCL9, E-cadherin, and vimentin in non-transduced (NT), control, and BCL9 KD DCIS.COM (A) (n = 4), and BCL9 and E-cadherin in NT, control, and BCL9 KD SUM225 (B) (n = 3). Data represent mean ± standard error of the mean (*P <0.05). (PDF 492 kb) [file 13058_2015_630_MOESM8_ESM.pdf]
