# Supplementary material for: Expression profiling of in vivo ductal carcinoma in situ progression models identified B cell lymphoma-9 as a molecular driver of breast cancer invasion
Source: Breast Cancer Res. 2015 Sep 17;17:128. doi: 10.1186/s13058-015-0630-z (PMC4574212; doi:10.1186/s13058-015-0630-z)

A.

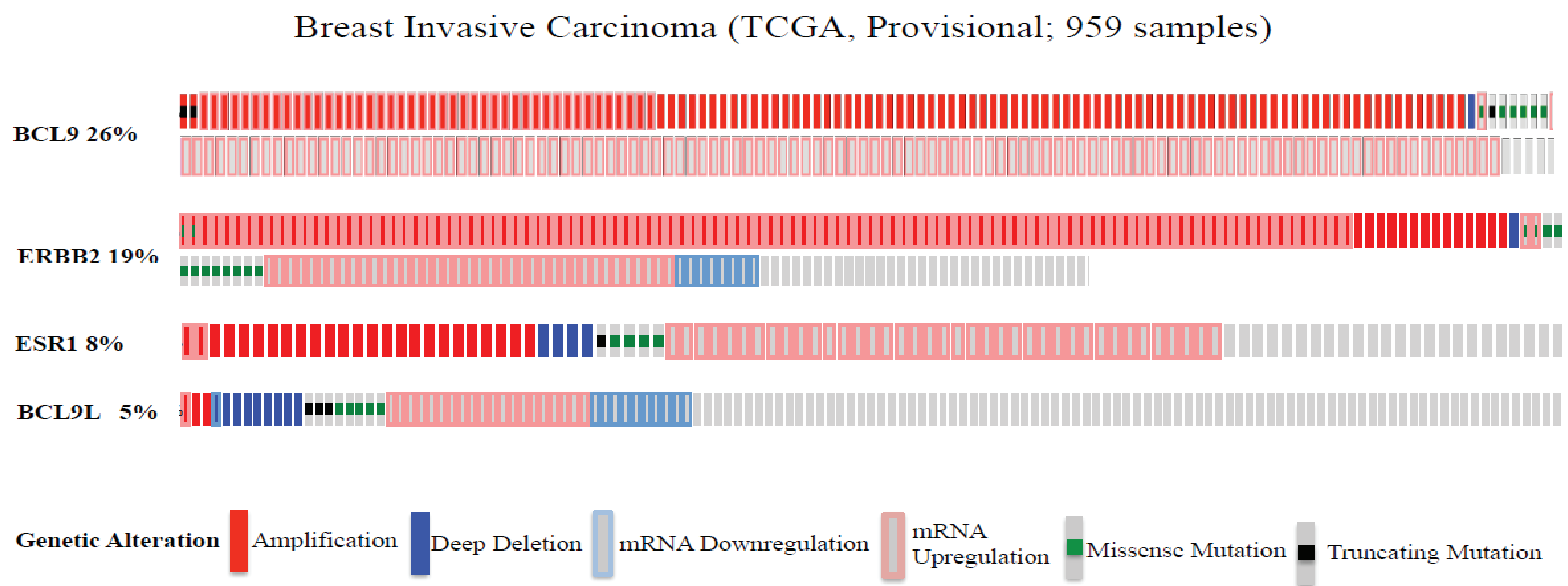

B.

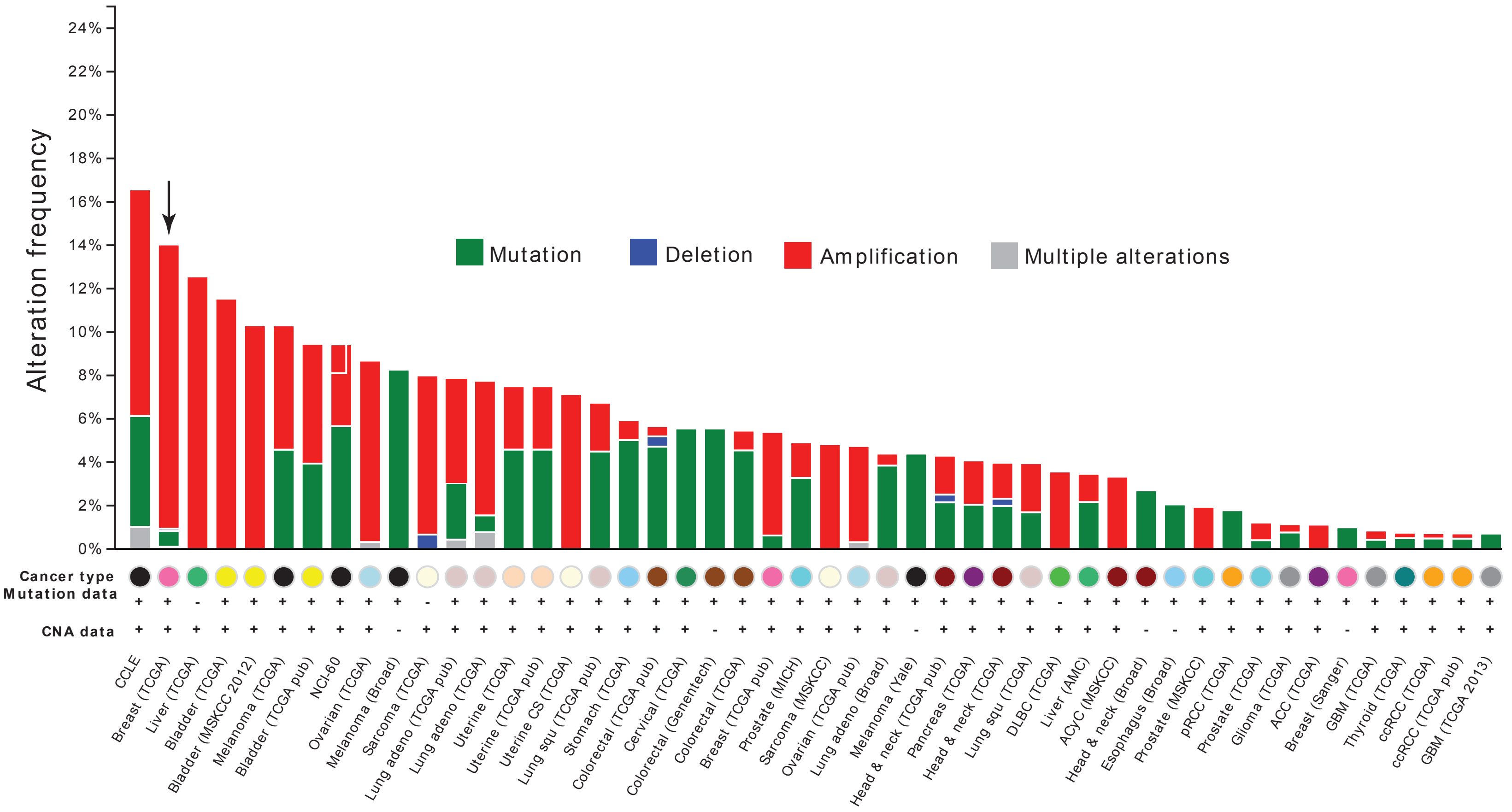

C.

	All tumors	*Basal*	*Luminal A*	*Luminal B*	*HER2 enriched*
No. Cases	825	81	235	133	58
No. Amplified	37	15	5	3	3
% Amplified	4.5%	18.5%	2.1%	2.3%	5.2%
95% CI	3.2%,6.1%	10.8%, 28.7%	0.7%, 4.9%	0.5%, 6.5%	1.1%, 14.4%

D.

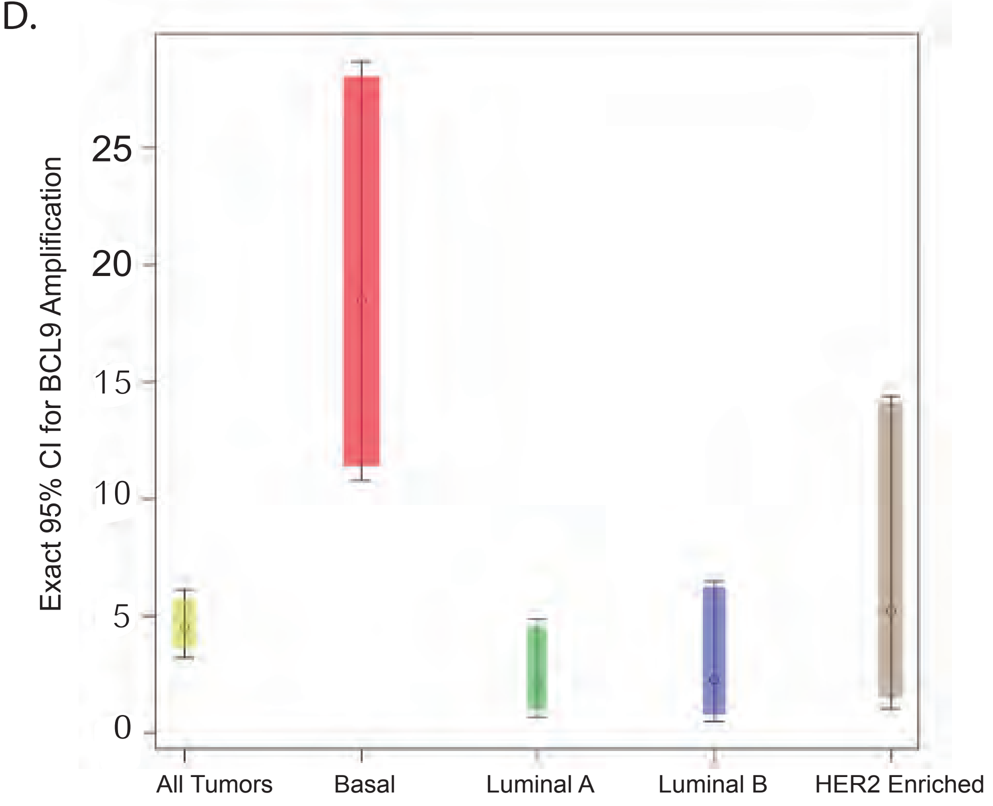

E.

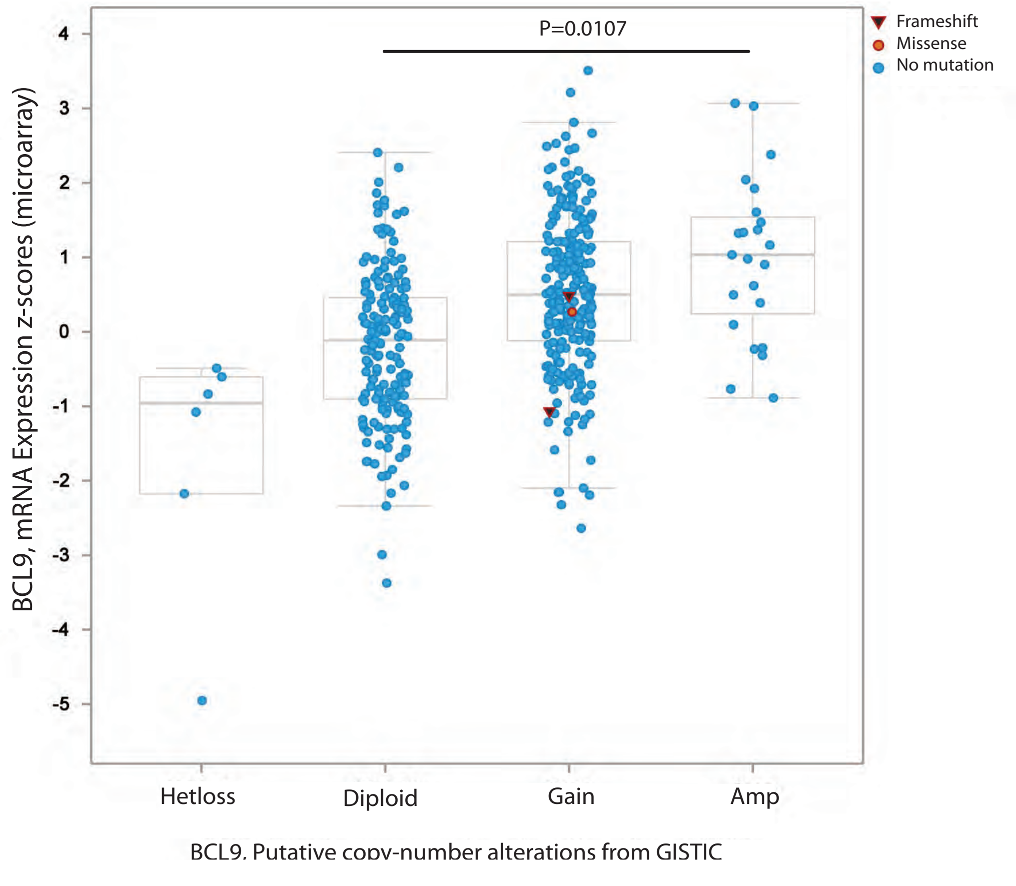

Supplement: Additional file 9: Figure S6. — A significant proportion of breast cancers showed BCL9 gene alteration. A The Cancer Genome Atlas (TCGA) provisional data showed that 26 % of invasive breast cancers (total of 965 cases) contained a genetic alteration in the BCL9 gene; the majority of these consisted of gene amplification and mRNA upregulation. This was a significant level of genetic alteration when compared to ERBB2 (19 %), ESR1 (8 %) and BCL9L (5 %). B BCL9 gene alterations across all cancers. The BCL9 gene was altered in many cancers including breast, liver and bladder. The arrow points to invasive breast cancers showing 14 % gene alteration (135 in 962 cases). C, D TCGA data showed that a significantly higher proportion of basal breast cancers contained BCL9 genomic amplification compared to the other subtypes (total of 825 cases). E The gene expression data were available as z scores. Diploid classification was used to identify median gene expression value, and this value was used as the cutoff for dichotomizing gene expression as low (<= median) or high (>median) for BCL9 expression. Contingency tables were created for diploid vs amplified or gain vs amplified against low or high BCL9 expression. Chi-square analysis indicated significant association between BCL9 amplification and high levels of BCL9 gene expression compared to diploid samples. (PDF 3138 kb) [file 13058_2015_630_MOESM9_ESM.pdf]
